# Palmitoylethanolamide supplementation for human health: A state-of-the-art systematic review of Randomized Controlled Trials in patient populations

**DOI:** 10.1016/j.bbih.2024.100927

**Published:** 2024-12-23

**Authors:** R. Bortoletto, C. Comacchio, M. Garzitto, F. Piscitelli, M. Balestrieri, M. Colizzi

**Affiliations:** a Unit of Psychiatry and Eating Disorders, Department of Medicine (DMED), University of Udine, Udine, Italy; b Institute of Biomolecular Chemistry, National Research Council (CNR), Pozzuoli, Italy; c Department of Psychosis Studies, Institute of Psychiatry, Psychology and Neuroscience, King's College London, London, UK

**Keywords:** Peroxisome proliferator-activated receptor α, Glutamate, Neuroinflammation, Nutraceutical, Endocannabinoidome, Cannabidiol

## Abstract

Interest in preventative dietary interventions for human health has increasingly focused on the endocannabinoid (eCB)-like compound palmitoylethanolamide (PEA), a bioactive lipid mediator with anti-inflammatory, analgesic, and neuroprotective properties. This Preferred Reporting Items for Systematic Reviews and Meta-Analyses (PRISMA) 2020-compliant systematic review aimed at collecting and comprehensively discussing all available data from Randomized Controlled Trials (RCTs) evaluating the efficacy and tolerability of PEA supplementation across human illnesses in patient populations. Overall, 48 eligible outputs from 47 RCTs were extracted, covering neuropsychiatric (*n* = 15), neurological (*n* = 17), somatic (*n* = 13), and visceral (*n* = 11) disturbances, as well as PEA effects on blood/plasma or other tissue biomarkers (*n* = 10). The strongest evidence emerged from RCTs exploring PEA impact on pain management and measures of general wellbeing, especially in its ultramicronized/micronized or cold-water dispersible formulations, showing good tolerability compared to controls. Also, alongside symptom improvement, PEA demonstrated to modulate biomarkers early altered in the initial phases of an illness or contributing to its progression, suggesting a disease-modifying potential. This systematic review provided a comprehensive overview of the therapeutic potential of PEA across RCTs, highlighting its versatility either as monotherapy or add-on treatment for various clinical conditions.

## Highlights

• Palmitoylethanolamide (PEA) is a safe anti-neuroinflammatory dietary supplement.
• 47 randomized controlled trials (RCTs) so far have studied PEA in clinical samples.
• Most RCTs show PEA efficacy on pain management and measures of general wellbeing.
• PEA ultramicronized/micronized formulations are among the most used and effective.
• Symptom improvement was often accompanied by the modulation of immune biomarkers.

## Graphical abstract

**Figure:**
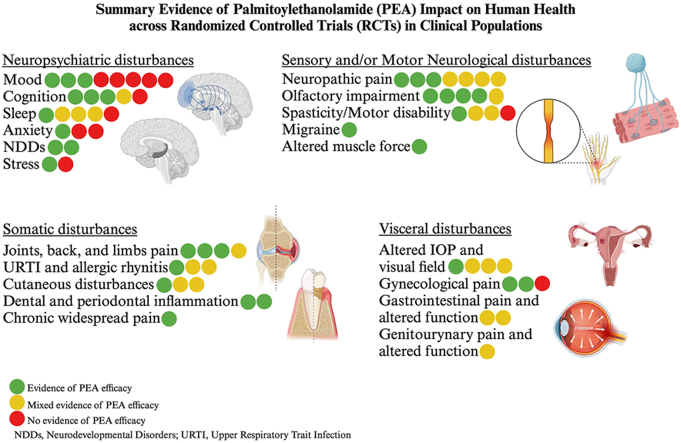

## 1 Introduction

Recent years have seen increasing research and social awareness around healthy nutrition and dietary habits as key factors in primary prevention (Robinson, 2018; Papadopoulou et al., 2023). This has contributed to promote public demand for nutraceuticals and dietary supplements as all-natural products, as well as their use in routine clinical practice, either as symptomatic treatments or as strategies with potential preventive properties especially targeting inflammatory processes (Chandra et al., 2022; Feart, 2020; Dwyer et al., 2018). Indeed, while inflammation is a natural response to remove harmful stimuli and promote tissue repair, an overly intense or prolonged inflammatory response can damage host tissues, cause organ dysfunction, and finally contribute to the medical burden in high-income countries (Skaper, 2017; Skaper et al., 2018). Concurrently, the search for novel pharmaceuticals, aimed at halting the progression of illnesses by intervening over their underlying mechanisms, has drawn growing attention to the endocannabinoid (eCB) system modulation, particularly due to its role in maintaining homeostasis during disease conditions as part of the enlarged family of lipid mediators known as the “endocannabinoidome” (eCBome) (Iannotti and Piscitelli, 2018; Veilleux et al., 2019). In addition to the two main eCBs, anandamide (AEA) and 2-arachidonoylglycerol (2-AG), which directly interact with cannabinoid receptors [i.e., type-1 (CB1), type-2 (CB2)], "eCB-like compounds" including the N-acylethanolamines (NAEs) palmitoylethanolamide (PEA) and oleoylethanolamide (OEA), and the monoacylglycerol (MAG) 2-oleoylglycerol (2-OG), have been extensively investigated for their analgesic and anorexigenic properties, as well as for their effect on lipid metabolism (Kleberg et al., 2014; Rahman et al., 2021; Tutunchi et al., 2023; Laleh et al., 2018; Shivyari et al., 2024; Kazemi et al., 2022; Mandoe et al., 2015). Undoubtedly, among eCB-like compounds, PEA use as a dietary supplement is the most widely studied across several clinical conditions, thanks to its analgesic, anti-neuroinflammatory, and neuroprotective features (Petrosino and Schiano Moriello, 2020; Bortoletto et al., 2022; Clayton et al., 2021a; Lang-Illievich et al., 2023). As a naturally occurring lipid, PEA is produced by different cell types to restore disrupted homodynamic balance in response to actual or potential damage, exerting its effects in multiple ways: (i) directly activating the Peroxisome Proliferator Activated Receptor-α (PPAR-α) and G Protein-coupled Receptor 55 (GPR55), subsequently activating the Transient Receptor Potential Vanilloid 1 (TRPV1) channels and increasing CB2 expression (Petrosino and Di Marzo, 2017; Lo et al., 2005; Pertwee, 2007); (ii) allosterically modulating the TRPV1, that interacts with CB1 and CB2 receptors (Petrosino and Di Marzo, 2017; Ambrosino et al., 2013); (iii) inhibiting the Fatty-Acid Amide Hydrolase (FAAH) and stimulating the Diacylglycerol Lipase (DAGL), thus increasing the endogenous availability of AEA and 2-AG and their actions at CB1, CB2 and TRPV1 receptors (Petrosino et al., 2016; Di Marzo et al., 2001); (Feart, 2020) down-regulating mast cell and microglia activation via an “Autacoid Local Inflammation Antagonism” (ALIA) effect (Petrosino et al., 2019; Scuderi et al., 2011). According to its pharmacodynamics, PEA is partially seen as the endogenous equivalent of the phytocannabinoid cannabidiol (CBD) (Clayton et al., 2021b), which has been thoroughly studied for its therapeutic potential in multiple neuropsychiatric disorders, chronic pain, and gastrointestinal motility issues, although being not devoid of adverse events (AEs) and raising increasing concern about possible interactions with other medications (Bonaccorso et al., 2019; Lattanzi et al., 2021; Villanueva et al., 2022; Story et al., 2023; Madeo et al., 2023). Alongside the safe and tolerable profile of exogenous PEA supplementation, the above considerations make it an advantageous alternative to CBD in the long-term treatment of both clinical and general populations, particularly in its most bioavailable formulations (Clayton et al., 2021a, 2021b; Clayton et al., 2021a, 2021b; Gabrielsson et al., 2016; Impellizzeri et al., 2014; Briskey et al., 2019).

### 1.1 Objectives

The anti-inflammatory properties of PEA may represent a promising therapeutic mechanism underlying its clinical utility across several human illness conditions, as both a symptomatic agent and a disease-modifying drug. This systematic review aims at collecting and discussing all available data generated from Randomized Controlled Trials (RCTs) exploring PEA efficacy and tolerability across all human illness conditions in clinical populations.

## 2 Materials and methods

### 2.1 Inclusion and exclusion criteria

All findings emerging from RCTs regarding the effects of PEA supplementation for human diseases in clinical populations were gathered and systematically analyzed according to the Preferred Reporting Items for Systematic Reviews and Meta-Analyses (PRISMA) 2020 guidelines (Parums, 2021). Since this systematic review was intended at collecting the highest level of evidence-based clinical data on PEA supplementation versus comparator treatments, only RCT designs were considered for systematic data extraction, due to their intrinsically lower susceptibility to biases (Burns et al., 2011). Patient populations suffering from all illness conditions studied through RCTs with PEA, evaluated using appropriate clinical assessments or assessment scales of targeted symptoms, of any age, ethnicity, and gender, were included. Exclusion criteria were as follows: (i) non-RCTs in which PEA was the intervention of interest; (ii) RCTs in which PEA was not the intervention of interest; (iii) RCTs in which PEA was administered to healthy populations; (iv) case reports, case series, letters to the editor, correspondences, and commentaries; (v) cross-sectional studies, case-control studies, and cohort studies; (vi) reviews, systematic reviews, and meta-analyses.

### 2.2 Search strategy

A literature search was conducted across both electronic publication repositories (PubMed, Scopus, and Web of Science) and clinical trial registries [ClinicalTrials.gov, World Health Organization (WHO) International Clinical Trials Registry Platform (ICTRP)]. The first search encompassed all English-written original results of eligible studies published up to May 3rd, 2023, without any restrictions in terms of publication date, study duration, and follow-up. A second, more updated search was performed on August 26th, 2024, following the same methodology. To be as much inclusive as possible, the search was divided as follows: (i) clinical trial registries ClinicalTrials.gov and ICTRP were queried using the search terms “palmitoylethanolamide” and “palmidrol”; (ii) PubMed was queried using the search string “(palmitoylethanolamide OR palmitylethanolamide OR N-2-hydroxyethyl-hexadecanamide OR N-2-hydroxyethyl-palmitate OR N-palmitoylethanolamine OR palmidrol OR impulsin OR um-pea)” and applying the ‘article type’ filters ‘Clinical Trial’ and ‘Randomized Controlled Trial’; (iii) publication repositories Pubmed, Web of Science, and Scopus were queried using a combination of broad-meaning terms describing and/or concerning intervention “(palmitoylethanolamide OR palmitylethanolamide OR N-2-hydroxyethyl-hexadecanamide OR N-2-hydroxyethyl-palmitate OR N-palmitoylethanolamine OR palmidrol OR impulsin OR um-pea)”, study type “(prospective OR randomised OR randomized OR trial OR observational OR rct OR (randomized AND controlled AND trial))”, and study population “(human OR female OR male OR proband OR patient∗ OR volunteer∗ OR healthy OR adult OR child)”.

### 2.3 Data extraction, screening, and risk of bias assessment

Data extraction and screening were conducted by using a web-based systematic review management software (Covidence systematic review software, Veritas Health Innovation, Melbourne, Australia).

Quality of studies assessment was conducted according to the Johanna Briggs Institute (JBI) critical appraisal tool for RCTs (Barker et al., 2023). The JBI tool for RCTs consists of a 13-item scale exploring the internal validity (e.g., randomized allocation, level of concealment), similarity of participants and treatments between compared groups, the reliability of outcome measures, and the appropriateness of statistical analysis. Each item was parsed using four evaluation answers: “Yes”, “No”, “Unclear”, and “Not Applicable”. Affirmative answers were summarized as a score from 0 to 13. RCTs with a score equal or lower than 5 were classified as having a poor quality, those with a score between 6 and 7 as having a fair quality, and those with a score equal or higher than 8 as having a good quality (Barker et al., 2023).

All these steps were performed by two researchers (R.B. and C.C.), independently of each other.

In the instances of conflicting opinions regarding papers' inclusion or quality assessment, a consensus was reached through discussion involving a third senior reviewer (M.C.).

The full study protocol (PROSPERO, 2023 CRD42023423617) is available at: https://www.crd.york.ac.uk/prospero/display_record.php?ID=CRD42023423617 (accessed on October 3rd, 2024).

## 3 Results

### 3.1 Study selection

**Fig. 1:**
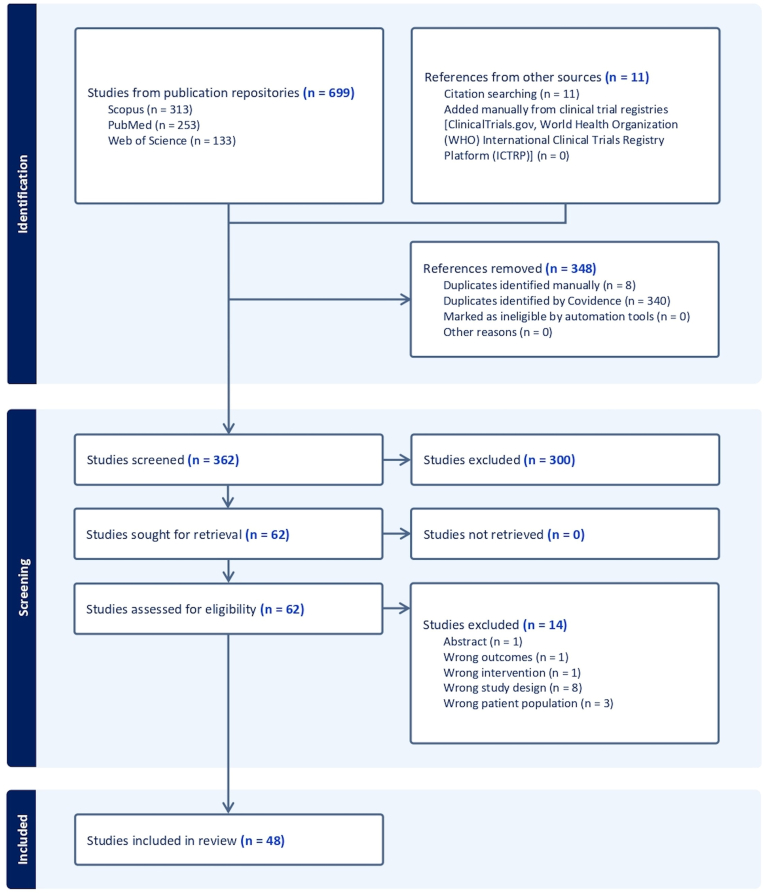
PRISMA flowchart of search strategy for systematic review.

Overall, 699 studies were imported from initial data search. After removing duplicates, 351 papers were retrieved. Titles, abstracts, full texts, and reference lists of all records were assessed against inclusion and exclusion criteria, according to a three-step screening approach (Fig. 1). A final list of 48 studies was used for systematic reappraisal, including forty-seven RCTs exploring PEA efficacy and tolerability across illness conditions. The identified RCTs explored the effect of PEA supplementation on a heterogeneous group of illnesses enclosing (i) neuropsychiatric disturbances (Abedini et al., 2022; Andresen et al., 2016; Campolo et al., 2021; Evangelista et al., 2018; Gagliano et al., 2011; Ghazizadeh-Hashemi et al., 2018; Khalaj et al., 2018; Lunardelli et al., 2019; Orefice et al., 2016; Pickering et al., 2022; Rao et al., 2021; Salehi et al., 2022; Steels et al., 2019; Versace et al., 2023; Bonzanino et al., 2024), (ii) sensory and/or motor neurological disturbances (Andresen et al., 2016; Campolo et al., 2021; Evangelista et al., 2018; Orefice et al., 2016; Pickering et al., 2022; Bonzanino et al., 2024; Coraci et al., 2018; D'Ascanio et al., 2021; Di Stadio et al., 2022; Di Stadio et al., 2023a; Faig-Marti and Martinez-Catassus, 2017; Faig-Marti and Martinez-Catassus, 2020; Guida et al., 2010; Ottaviani et al., 2019; Palma et al., 2016; Cantone et al., 2024; Di Stadio et al., 2023b; Briskey et al., 2024), (iii) somatic disturbances (Orefice et al., 2016; Steels et al., 2019; Bacci et al., 2011; Germini et al., 2017; Isola et al., 2021; Marini et al., 2012; Masek et al., 1974; Yuan et al., 2014; Briskey et al., 2023; Kadanangode et al., 2023; Rao et al., 2023a, 2023b; Salaffi et al., 2023), (iv) visceral disturbances (Gagliano et al., 2011; Cobellis et al., 2011; Cremon et al., 2017; Giammusso et al., 2017; Murina et al., 2013; Rossi et al., 2020; Strobbe et al., 2013; Tartaglia et al., 2015; Costagliola et al., 2014; Di Nardo et al., 2024), and (v) blood/plasma or other tissue biomarkers alterations (Orefice et al., 2016; Pickering et al., 2022; Rao et al., 2021; Steels et al., 2019; Isola et al., 2021; Briskey et al., 2023; Kadanangode et al., 2023; Cobellis et al., 2011; Cremon et al., 2017; Albanese et al., 2022). Moreover, in some cases the effect of PEA was evaluated in terms of quality of life due to symptom management (Orefice et al., 2016; Pickering et al., 2022; Rao et al., 2021; Steels et al., 2019; Guida et al., 2010; Germini et al., 2017; Masek et al., 1974; Rao et al., 2023a; Rao et al., 2023b; Salaffi et al., 2023; Giammusso et al., 2017; Rossi et al., 2020). A brief synthesis of the results is presented below and summarized in Table 1.

**Table 1:** Summary results of Randomized Controlled Trials investigating Palmitoylethanolamide supplementation across human diseases.

Study ID	Aim of study	Type of study	Sample size	Outcome measures	Summary outcomes
Abedini et al., 2022 (Iran)	To assess PEA effect on acute mania in BPAD patients	PEA effect on mood, anxiety, and stress	63 (PEA: 32; PLB: 31)	1.Neuropsychiatric measurement (YMRS, HDRS) 2.AEs measurement (ESRS, open-ended questions, comprehensive side effect checklist)	1.Neuropsychiatric measurement: (a) **YMRS: time × treatment interaction**; **YMRS global score**: PEA vs. PLB, NS (Baseline, Week 2, Week 4); **PEA < PLB (Week 6)**; **YMRS score changes**: From Baseline to Week 2: PEA vs. PLB, NS; **From Baseline to Week 4, From Baseline to Week 6: PEA > PLB**; (b) HDRS score changes: From Baseline to Week 6: PEA vs. PLB, NS 2.AEs measurement: (a) ESRS: time × treatment interaction, NS; ESRS global score: PEA vs. PLB, NS (Baseline, Week 1, Week 2, Week 4, Week 6); ESRS score changes: all comparisons, NS; (b) Frequency of adverse events (drowsiness, dizziness, increased appetite, skin rash, diarrhea, dry mouth, sore throat, tachycardia): PEA vs. PLB, NS
Albanese et al., 2022 (Italy)	To assess PEA effect on inflammatory response in COVID-19 patients	PEA effect on blood/plasma or other tissue biomarkers alterations	90 (PEA: 45; CTRL: 45)	Blood/plasma and/or other tissue measurement (Inflammatory markers quantification, oxidative stress markers quantification)	**D-Dimer, CRP, IL-6, CD3 + CD8 + absolute count, Anti-SARS-CoV-2 IgG, Lymphocytes, Neutrophil/Lymphocytes ratio (T1-T0): PEA > CTRL**; Red blood cell, Hemoglobin, Hematocrit, MCV, Neutrophil, PLT, Myoglobin, PT, Fibrinogen, Antithrombin III, Creatinine, GFR, Nitrogenic Acid, D Vitamin, ESR, TNF-α, Liver function, Muscle damage: PEA vs. CTRL, NS; ΔFORD(T1-T0): PEA vs. CTRL, NS; **ΔFORT(T1-T0): PEA < CTRL**
Andresen et al., 2016 (Denmark, Norway, United Kingdom)	To assess PEA effect on neuropathic pain and spasticity in SCI patients	1. PEA effect on mood, anxiety, and stress 2. PEA effect on sleep 3. PEA effect on neuropathic pain 4. PEA effect on mobility, spasticity, and sensory-motor deficits	73 (PEA: 36; PLB: 37)	1. Neurological measurement (NRS, use of rescue medication, neuropathic pain descriptors, evoked pain, MTS, PGIC, NNT) 2. Neuropsychiatric measurement (ISI, MDI, GAD-10) 3. QoL measurement (S-TOPS) 4. AEs measurement (Clinical assessment)	1. Neurological measurement: (a) ITT, secondary ITT, PP populations: PEA vs. PLB, NS; (b) **Use of rescue medication: PEA, ↓**; **PLB, ↑**; **treatment effect**; (c) **Self-reported spasticity: PEA, ↑**; **PLB, ↓**; **Δ(baseline- LWT): treatment effect**; (d) Stiffness, (e) Spasms, (f) Spasticity angle, (g) NPSI, (h) CPSS, (i) Unpleasantness, (j) Pain interference, (k) Sensory examination, (l) Global impression, (m) Pain relief of neuropathic pain, (n) Pain relief at-/below-level of neuropathic pain: PEA vs. PLB, NS (all comparisons) 2. Neuropsychiatric measurement: (a) Anxiety, (b) Depression, (c) Sleep disturbance, (d) Insomnia: PEA vs. PLB, NS (all comparisons) 3. S-TOPS: PEA vs. PLB, NS 4. AEs measurement: PEA vs. PLB, NS
Bacci et al., 2011 (Italy)	To assess PEA effect on swelling and pain in ILTM surgery patients	PEA effect on dental and periodontal inflammation	30 (PEA: 30; CTRL: 30)	1. Somatic measurement (Trismus, swelling, VAS, NSAIDs consumption) 2. AEs measurement (Clinical assessment, post-operative complications)	1. Somatic measurement: (a) **Effect on mean VAS: time; treatment**; T0: PEA vs. CTRL, NS; **T1, T2: PEA < CTRL**; (b) **Effect on mouth opening range: time**; treatment, NS; **gender**; T0, T1, T2: PEA vs. CTRL, NS; (c) Effect on distance from lateral canthus to gonion: all comparisons, NS; (d) **Effect on distance from labial commissure to tragus: time**; treatment, NS; T0, T1, T2: PEA vs. CTRL, NS 2. AEs measurement: PEA vs. PLB, NS
Bonzanino et al., 2024 (Italy)	To assess PEA effect on neurological deficit, independence, disability, and cognitive impairment in AIS patients	1. PEA effect on attention, awareness, and cognition 2. PEA effect on mobility, spasticity, and sensory-motor deficits	60 (PEA: 30; CTRL: 30)	1. Neurological assessment (11-item NIHSS, Barthel index, mRS) 2. Neuropsychiatric measurement (MMSE, MoCA) 3. AEs measurement (Clinical assessment)	1. Neurological measurement: (a) **NIHSS score: T2 < T1 < T0 (PEA, CTRL)**; PEA vs. CTRL, NS; (b) **Barthel index: T2 > T1 > T0 (PEA, CTRL)**; PEA > CTRL, NS (T2); PEA vs. CTRL, NS (T0, T1); (c) **mRS score: T2 < T1 < T0 (PEA, CTRL)**; **PEA < CTRL (T2)**; PEA vs. CTRL, NS (T0, T1) 2. Neuropsychiatric measurement: (a) number of patients able to perform MMSE, (b) number of patients able to perform MoCA: PEA > CTRL, NS (T1, T2); (c) MMSE mean score, (d) MoCA mean score: T2 > T1, NS (PEA) 3. AEs measurement: PEA vs. CTRL, NS
Briskey et al., 2023 (Australia)	To assess PEA effect on allergy symptoms in AR patients	1. PEA effect on respiratory tract infections and allergy symptoms 2. PEA effect on blood/plasma or other tissue biomarkers alterations	108 (PEA: 54; PLB: 54)	1. Somatic measurement (rTNSS, RQLQ) 2. Blood/plasma and/or other tissue measurement (Inflammatory markers quantification, full blood count, enzyme and liver function test)	1. Somatic measurement: (a) rTNSS score: PEA vs. PLB, NS (all timepoints); (b**) rTNSS morning score: ↓ from Day 3 (PEA, PLB)**; (c) **rTNSS evening score: ↓ from Day 2 (PEA, PLB)**; (d) RQLQ score: PEA vs. PLB, NS; (e) **rTNSS score (sub-group with rTNSS > 4): Week 2 < baseline, Week 1**; (f) RQLQ total score: PEA vs. PLB, NS; (g) **RQLQ (all domains): ↓ overtime (PEA, PLB)** 2. Blood/plasma and/or other tissue measurement: (a) **histamine levels change: PEA > PLB**; (b) **IL-4 levels: Week 2 < baseline (PEA, PLB)**; (c) **IL-10 levels**, (d) **IL-8 levels**, (e) **TNF-α levels**, (f) **histamine levels: Week 2 < baseline (PEA)**; Week 2 vs. baseline, NS (PLB)
Briskey et al., 2024 (Australia)	To assess PEA effect on pain and symptom duration in migraine patients	PEA effect on pain associated with migraine	80 (PEA: 40; PLB: 40)	1. Neurological measurement (VAS, migraine duration, migraine severity, use of rescue medication) 2. AEs measurement (Clinical assessment, self-administered questionnaire)	1. Neurological measurement: (a) number of migraines, (b) severity of migraines at onset, (c) use of rescue medication at 4 h: PEA vs. PLB, NS; (d) **number of resolved migraines at 2 h**, (e) **number of resolved migraines at 2 h with no use of rescue medication**, (f) **number of resolved moderate-at-onset migraines**, (g) **number of resolved migraine events at 8 h: PEA > PLB**; (h) **number of unresolved migraine events at 8 h**, (i) **number of reported rescue medication use: PEA < PLB**; (j) **VAS score: 30 min < baseline (PLB)**; **60 min < baseline (PEA)**; PEA vs. PLB, NS (all timepoints); (k) **VAS score change: PEA > PLB (1.5 h, 4 h)**; PEA vs. PLB, NS (other timepoints) 2. AEs measurement: PEA vs. PLB, NS
Campolo et al., 2021 (Italy)	To assess PEA effects on memory and cognitive function in TBI patients	1. PEA effect on mood, anxiety, and stress 2. PEA effect on attention, awareness, and cognition 3. PEA effect on mobility, spasticity, and sensory-motor deficits	30 (PEA: 15; CTRL: 15)	1. Neuropsychiatric measurement (GCS, Marshal Score, MMSE, BNCE, BDI) 2. Neurological measurement (Barthel index)	1. Neuropsychiatric measurement: (a) **MMSE score: PEA > CTRL**; **ΔMMSE: PEA > CTRL**; **MMSE score > 26: PEA > CTRL**; (b) BNCE: PEA vs. CTRL, NS; **BNCE Interference Memory: PEA > CTRL**; (c) BDI: PEA vs. CTRL, NS 2. Independency (**Barthel index**): **↑ PEA vs. baseline**; PEA vs. CTRL, NS
Cantone et al., 2024 (Italy)	To assess PEA effect on olfactory function recovery in post-COVID-19 olfactory impairment	PEA effect on altered olfactory function	89 (Co-ultraPEALut: 17; ALA: 21; Co-ultraPEALut/ALA: 28; PLB: 23)	Neurological measurement (Sniffin' sticks)	**TDI score:** PLB post vs. PLB pre, NS; ALA post vs. ALA pre, NS; **Co-ultraPEALut post > Co-ultraPEALut pre**; **Co-ultraPEALut/ALA post > Co-ultraPEALut/ALA pre**; **TDI score at T0: PLB > Co-ultraPEALut, Co-ultraPEALut/ALA, ALA**; other comparisons, NS; **TDI score at T1: Co-ultraPEALut > PLB, ALA; Co-ultraPEALut/ALA > ALA;** other comparisons, NS **Parosmia resolution rates: Co-ultraPEALut > ALA**; **Co-ultraPEALut/ALA > Co-ultraPEALut, ALA, PLB**; other comparisons, NS
Cobellis et al., 2011 (Italy)	To assess PEA effect on chronic pelvic pain in EMS patients	1. PEA effect on gynecological and genitourinary pain or altered function 2. PEA effect on blood/plasma or other tissue biomarkers alterations	61 (PEA: 21; PLB: 20; Celecoxib: 20)	1. Visceral measurement (Pelvic examination, transvaginal US, symptom questionnaire, VAS) 2. Blood/plasma and/or other tissue measurement (Inflammatory markers quantification) 3. AEs measurement (Clinical assessment)	1. Visceral measurement: (a) **Dysmenorrhea,** (b) **Dyspareunia,** (c) **Pelvic Pain: 3 months vs. baseline, ↓ (all groups); Celecoxib < PEA < PLB (at 3 months)** 2. Blood/plasma measurement: (a) ESR, (b) CRP levels: PEA vs. Celecoxib vs. PLB, NS 3. AEs measurement: PEA vs. Celecoxib vs. PLB, NS
Coraci et al., 2018 (Italy)	To assess PEA effect on pain relief in CTS patients	PEA effect on neuropathic pain	56	Neurological measurement (Mean I and III digits SNCV and SNCA, III digit mean SAP, US W/F cross sectional area ratio of the median nerve, BCTQ)	**Mean SNCV III finger (ΔT)**, **Mean SAP III finger (ΔT): PEA group, ↑**; **PLB group, ↓**; T1 vs. T0, NS (both groups); **Mean US W/F (ΔT): PEA group, ↓**; **PLB, group, ↑**; T1 vs. T0, NS (both groups)
Costagliola et al., 2014 (Italy)	To assess PEA effect on IOP and visual field in NTG patients	PEA effect on intraocular pressure and visual field	32 (PEA: 16; CTRL: 16)	1. Visceral measurement (BCVA, IOP with patient in sitting position at the slit lamp, CCT, visual field, red-free disc photo) 2. AEs measurement (Self-administered questionnaire)	1. Visceral measurement: (a) **IOP**, (b) **visual field (MD, PSD): Month 6 < baseline (PEA)**; Month 6 vs. baseline, NS (CTRL); (c) BCVA changes: PEA, NS; CTRL, NS 2. AEs measurement: PEA vs. CTRL, NS
Cremon et al., 2017 (Italy, Spain, France, Croatia, Bosnia)	To assess PEA effect on mast cell count/activation and symptoms in IBS patients	1. PEA effect on gastrointestinal pain or altered function 2. PEA effect on blood/plasma or other tissue biomarkers alterations	54 (PEA: 29; PLB: 25)	1. Visceral measurement (Symptom questionnaire) 2. Blood/plasma and/or other tissue measurement (Inflammatory markers quantification, Immunohistochemistry, EIA, NGF Emax ImmunoAssay System, ELISA; eCBs/NAEs quantification, LC-APCI-MS; protein expression, Western blot) 3. AEs measurement (Clinical assessment)	1. Visceral measurement: (a) **Effect on abdominal pain/discomfort severity: time, ↓; treatment, ↓; treatment × time interaction, ↓**; responding patients: PEA vs. PLB, NS; (b) **Effect on abdominal pain/discomfort frequency: time, ↓**; **treatment, ↓**; treatment × time interaction, NS; Effect on (c) bloating severity and frequency, (d) bowel habit, (e) dyspeptic and gastro-esophageal reflux symptoms: treatment × time interaction, NS; (f) **Effect on general well-being: time, ↑**; treatment × time interaction, NS 2. Blood/plasma measurement: (a) **Effect on MC count over time: IBS > HC**; treatment, NS; gender, NS; centre, NS; bowel presentation, NS; time, NS; (b) Effect on immune cell/MC activation: IBS vs. HC, NS; treatment, NS; (c) **Effect on eCBs/AEs: OEA levels: IBS < HC**; **CB2 expression: IBS > HC**; AEA, 2-AG, PEA, OEA association with bowel habit: NS; **CB1 expression, FAAH levels: IBS-C > IBS-D, IBS-M**; treatment, NS 3. AEs measurement: PEA vs. PLB, NS
D'Ascanio et al., 2021 (Italy)	To assess PEA effect on olfactory function recovery in post-COVID-19 olfactory impairment	PEA effect on altered olfactory function	12 (PEA: 7; CTRL: 5)	Neurological measurement (Sniffin' sticks)	**Sniffin' Sticks score improvement (T1-T0): PEA > CTRL**
Di Nardo et al., 2024 (Italy)	To assess PEA effect on pain and other symptoms in IBS patients	PEA effect on gastrointestinal pain or altered function	70 (PEA: 34; PLB: 36)	1. Visceral measurement (IBS-SSS, BSS) 2. AEs measurement (Direct interview)	1. Visceral measurement: (a) **number of complete remissions after 12 weeks (IBS-SSS < 75): PEA > PLB (IBS-D)**; PEA vs. PLB (IBS-C, IBS-M); (b) **abdominal pain frequency (from baseline to Week 12): PEA, ↓**; PLB, NS; (c) **total IBS-SSS**, (d) **pain intensity score**, (e) **life interference score (from baseline to Week 12): PEA, ↓; PLB, ↓;** (f) **total IBS-SS (Week 12)**, (g) **pain intensity score (Week 8, Week 12)**, (h) **pain frequency score (Week 8, Week 12): PEA < PLB**; (i) bowel habit changes: all comparisons, NS 2. AEs measurement: PEA vs. PLB, NS
Di Stadio et al., 2022 (Italy)	To assess PEA effect on olfactory function recovery in post-COVID-19 COD	PEA effect on altered olfactory function	185 (PEA: 130; PLB: 55)	1. Neurological measurement (Sniffin' Sticks) 2. AEs measurement (Clinical assessment)	1. Neurological measurement: (a) **TDI score: PEA post > PEA pre, PLB post**; PLB post vs. PLB pre, NS; PEA pre vs. PLB pre, NS; (b) **TDI Sniffin' Score: PEA post > PLB post**; PEA pre vs. PLB pre, NS; (c) **TDI score recovery: PEA > PLB**; (d) **TDI score variation (PEA group): T3 > T0, T1**; **T0 > T2**; other comparisons, NS 2. AEs measurement: PEA vs. PLB, NS
Di Stadio et al., 2023a (Italy)	To assess PEA effect on olfactory function recovery in post-COVID-19 olfactory impairment	PEA effect on altered olfactory function	130 (PEA: 94; PLB: 36)	Neurological measurement (Sniffin' sticks)	**Sniffin’ Sticks score: PEA post > PEA pre, PLB post**; PLB post vs. PLB pre, NS; PEA pre vs. PLB pre, NS **Recovered anosmia patients at T1: PEA > PLB** Recovered parosmia patients at T1: PEA vs. PLB, NS
Di Stadio et al., 2023b (Italy)	To assess PEA effect on olfactory function recovery in post-COVID-19 olfactory impairment	PEA effect on altered olfactory function	250 (PEAdaily: 50; PEAbid: 50; PEA + OT: 100; PLB + OT: 50)	Neurological measurement (Sniffin' sticks)	**Sniffin’ Sticks score (≥ 3 points improvement, between-groups): PEA + OT > PEAdaily, PEAbid, PLB + OT** **Sniffin’ Sticks score (≥ 3 points improvement, within-group): PLB + OT, PEAdaily, PEAbid: Month 3 > baseline**; other comparisons, NS; **PEA + OT: Month 3 > Month 2 > Month 1 > baseline** **Sniffin’ Sticks score:** all comparisons, NS (baseline, Month 1); **PEA + OT, PEAdaily, PEAbid > PLB + OT (Month 2, Month 3)**; other comparisons, NS
Evangelista et al., 2018 (Italy)	To assess PEA effect on neuropathic pain intensity and sleep quality in CTS patients	1. PEA effect on sleep 2. PEA effect on neuropathic pain	42 (PEA: 22; CTRL: 20)	1. Neuropsychiatric measurement (NRS, PSQI, PGIC) 2. Neurological measurement (NRS, Tinel's sign, Phalen's test) 3. AEs measurement (Clinical assessment)	1. Neuropsychiatric measurement: (a) **Sleep-wake rhythm (NRS)**: PEA vs. CTRL, NS (baseline, 90 days post-surgery); **PEA < CTRL (60 days pre-surgery)**; (b) **PSQI Total**, (c) **PSQI Sleep latency**, (d) **PSQI Sleep disturbances**, (e) **PSQI Overall sleep quality**, (f) **PSQI Troubles in daily activities**: PEA vs. CTRL, NS (baseline, 90 days post-surgery); **PEA < CTRL (60 days pre-surgery)**; (g) **PSQI Sleep duration**: PEA vs. CTRL, NS (baseline, 90 days post-surgery); **PEA > CTRL (60 days pre-surgery)**; (h) **PGIC: PEA > CTRL** 2. Neurological measurement: (a) **Hyperalgesia score [NRS, Δ(surgery-baseline)]: PEA > CTRL**; (b) **Wrist** flexion **mean time [Whalen's test, Δ(surgery-baseline)]: PEA > CTRL** 3. AEs measurement: PEA vs. CTRL, NS
Faig-Marti and Martinez-Catassus, 2017 (Spain) Faig-Marti and Martinez-Catassus, 2020 (Spain)	To assess PEA effect on clinical and electrophysiological changes in CTS patients	PEA effect on neuropathic pain	61 (PEA: 30; PLB: 31)	1. Neurological measurement (ENG, Levine's. questionnaire, SSS, FSS, Durkan's test, Phalen's test, VAS) 2. AEs measurement (Clinical assessment)	1. Neurological measurement: (a) Durkan's test, (b) Phalen's test, (c) ENG data: PEA vs. PLB, NS; (d) Sensitive speed, (e) Sensitive peak amplitude, (f) Motor latency: PLB post-treatment vs. PLB pre-treatment, NS; (g) VAS: PEA vs. PLB, NS; PLB pre-treatment vs. PLB post-treatment, NS; (h) **FSS: PEA pre-treatment > PEA post-**treatment; PLB pre-treatment vs. PLB post-treatment, NS; (i) **SSS: PEA pre-treatment > PEA post-treatment**; **PLB pre-treatment > PLB post-treatment**; (j) **Levine's questionnaire: PLB post-treatment < PLB pre-treatment** 2. AEs measurement: PEA vs. PLB, NS
Gagliano et al., 2011 (Italy)	To assess PEA effect on IOP in POAG/OH patients	1. PEA effect on mood, anxiety, and stress 2. PEA effect on sleep 3. PEA effect on intraocular pressure and visual field	42 (PEA: 21; PLB: 21)	1. Visceral measurement (BCVA, anterior and posterior segment findings, vertical and horizontal C/D ratio, Goldmann applanation tonometry, visual field) 2. Neuropsychiatric measurement (MDQ) 3. AEs measurement (Questionnaire)	1. Visceral measurement: (a) **ΔIOP: PEA, ↓**; PLB, NS; (b) **IOP (1 month, 2 months): PEA < PLB**; (c) ΔBCVA, (d) ΔCCT, (e) vertical C/D ratio, (f) ΔMD, (g) ΔPSD: PEA, NS; PLB, NS 2. Neuropsychiatric measurement (ΔMDQ): PEA, NS; PLB, NS 3. AEs measurement: PEA vs. PLB, NS
Germini et al., 2017 (Italy)	To assess PEA effect on chronic pain in geriatric patients	PEA effect on joints, back, limbs, and chronic widespread pain and altered function	10 (PEA: 5; PLB: 5)	1. Somatic measurement (11-point VNS) 2. QoL measurement (BPFS) 3. AEs measurement (Clinical assessment)	1. Somatic measurement (**Effect on pain intensity**): **PEA > PLB (patient 2, patient 9)**; **PEA < PLB (patient 3, patient 5)**; PEA vs. PLB, NS (other patients 2. QoL measurement (**Effect on function impairment**): **PEA > PLB (patient 7)**; PEA vs. PLB, NS (other patients) 3. AEs measurement: PEA vs. PLB, NS
Ghazizadeh-Hashemi et al., 2018 (Iran)	To assess PEA effect on depressive symptoms in MDD patients	PEA effect on mood, anxiety, and stress	54 (PEA: 27; PLB: 27)	1. Neuropsychiatric measurement (HDRS) 2. AEs measurement (Side-effect checklist, self-reports)	1. Neuropsychiatric measurement: (a) **Effect on HDRS scores: time, ↓**; **treatment, ↓**; **time × treatment interaction, ↓**; (b) **Reduction in HDRS scores: PEA > PLB (week 2, week 4, week 6)**; (c) **HDRS improvement: PEA > PLB (week 6)**; (d) **HDRS score male subgroup (week 6): PEA < PLB**; (e) HDRS score female subgroup (week 6): PEA vs. PLB, NS; (e) suicide ideation: PEA vs. PLB, NS (baseline, week 6); (f) **Number of responders: PEA > PLB (week 6)**; PEA vs. PBL, NS (other timepoints); (g) **Number of remissions: PEA > PLB (week 6)**; PEA vs. PLB, NS (other timepoints) 2. AEs measurement (Frequency of adverse events): PEA vs. PLB, NS (daytime drowsiness, morning drowsiness, dizziness, slowed movement, tremor, increased appetite, nervousness, restlessness, skin rash, blurred vision, loss of appetite, fatigue, diarrhea, dry mouth, sore throat, tachycardia)
Giammusso et al., 2017 (Italy)	To assess PEA effect on chronic pain in CP/CPPS patients	PEA effect on gynecological and genitourinary pain or altered function	44	1. Visceral measurement (NIH/CPSI, IIEF5, IPSS) 2. QoL measurement (NIH/CPSI) 3. AEs measurement (Clinical assessment)	1. Visceral measurement, 2. QoL measurement: (a) IPSS score pre-treatment: PEA vs. CTRL, NS; (b) **IPSS score post-treatment: PEA < CTRL**; (c) IIEF5 score pre-treatment, (d) IIEF5 score post-treatment: PEA vs. CTRL, NS; (e) **NIH/CPSI score: post-PEA < pre-PEA**; pre-CTRL vs. post-CTRL, NS; other comparisons, NS 3. AEs measurement: PEA vs. CTRL, NS
Guida et al., 2010 (Italy)	To assess PEA effect on chronic neuropathic pain in compressive-type lumbar sciatica patients	PEA effect on neuropathic pain	636 (PEA: 427; PLB: 209)	1. Neurological measurement (VAS) 2. QoL measurement (RDQ) 3. AEs measurement (Clinical assessment)	1. Neurological measurement: (a) VAS baseline values: PEA(600 mg) vs. PEA(300 mg) vs. PLB, NS; (b) **ΔVAS(T21-baseline) values: PLB < PEA(300 mg) < PEA(600 mg)**; (c) **↑ Perceived efficacy, ↑ PEA dosage** 2. QoL measurement: (a) RDQ baseline values: PEA600 vs. PEA300 vs. PLB, NS; (b) **ΔRDQ(T21-baseline) values: PLB < PEA(300 mg) < PEA(600 mg)** 3. AEs measurement: PEA vs. PLB, NS
Isola et al., 2021 (Italy)	To assess PEA effect on pain and inflammatory response in periodontitis patients	1. PEA effect on dental and periodontal inflammation 2. PEA effect on blood/plasma or other tissue biomarkers alterations	66 (PEA: 33; CTRL: 33)	1. Somatic measurement (PD, BOP, VAS, PI, CAL, GR) 2. Blood/plasma and/or other tissue measurement (Inflammatory markers quantification) 3. AEs measurement (Clinical assessment)	1. Somatic measurement: (a) **PD: 180 days < baseline (PEA)**; 180 days vs. baseline, NS (CTRL); **PEA < CTRL (30, 60, 180 days)**; (b) **BOP: 180 days < baseline (both groups)**; **PEA < CTRL (15, 60, 180 days)**; (c) **PI: 180 days < baseline (CTRL)**; 180 days vs. baseline, NS (PEA); (d) **CAL:** PEA **> CTRL (30, 60 days)**; (e) **GR: PEA > CTRL (30, 60 days)**; (f) peak post-operative VAS score: 2h (PEA); 12h (CTRL); (g) **median VAS score: PEA < CTRL (6, 12, 24 h)** 2. Other tissue measurement: (a) **GCF volume: 180 days < baseline (both groups)**; (b) **IL-1β levels: PEA < CTRL (15, 30, 60 days)**; (c) **IL-10 levels:** PEA **> CTRL (30, 60 days)**; (d) **TNF-α levels: PEA < CTRL (30 days)**; (e) **IL-1β/IL-10 ratio: PEA < CTRL (60, 180 days)** 3. AEs measurement: PEA vs. CTRL, NS
Kadanangode et al., 2023 (India)	To assess PEA effect on joint pain and inflammatory response in arthritis patients	1. PEA effect on joints, back, limbs, and chronic widespread pain and altered function 2. PEA effect on blood/plasma or other tissue biomarkers alterations	72 (PEA: 36; CTRL: 36)	1. Somatic measurement (WOMAC) 2. Blood/plasma and/or other tissue measurement (Inflammatory markers quantification) 3. AEs measurement: Clinical assessment	1. Somatic measurement: (a) **pain score (WOMAC)**, (b) **joint stiffness (WOMAC)**, (c) **physical stiffness (WOMAC)**: baseline vs. Week 12, NS (CTRL); **Week 12 < baseline (PEA)** 2. Blood/plasma and/or other tissue measurement: (a) **TNF-α levels**, (b) **IL-1β levels**, (c) **IL-6 levels**, (d) **CRP levels:** baseline vs. Week 12, NS (CTRL); **Week 12 < baseline (PEA)** 3. AEs measurement: PEA vs. CTRL, NS
Khalaj et al., 2018 (Iran)	To assess PEA effect as on language and behavior in ASD patients	PEA effect on psychotic symptoms and autistic behaviors	62 (PEA: 31; PLB: 31)	1. Neuropsychiatric measurement (ABC-C) 2. AEs measurement (Clinical assessment)	1. Neuropsychiatric measurement: (a) **ABC-C over time: PEA < PLB (irritability and hyperactivity)**; PEA vs. PLB, NS (other domains); (b) **ABC-C at 10 weeks: PEA < PLB (irritability, hyperactivity)**; PEA < PLB, trend effect (inappropriate speech); PEA vs. PLB, NS; (c) **ABC-C at 5 weeks: PEA < PLB (hyperactivity)**; PEA < PLB, trend effect (stereotypic behavior, inappropriate speech); PEA vs. PLB, NS (other domains); (d) **ABC-C response rate at 10 weeks: PEA > PLB (hyperactivity, irritability, inappropriate speech)**; PEA vs. PLB, NS (other domains); (e) ABC-C response rate at 5 weeks: PEA vs. PLB, NS (all domains) 2. AEs measurement: PEA vs. PLB, NS
Lunardelli et al., 2019 (Italy)	To assess PEA effect on POD in hip fractured patients	PEA effect on attention, awareness, and cognition	80 (PEA: 40; CTRL: 40)	1. Neuropsychiatric measurement (DOM scale) 2. AEs measurement (Clinical assessment)	1. Neuropsychiatric measurement: (a) **POD incidence: PEA < CTRL**; (b) **Reduction in POD severity: PEA > CTRL**; (c) POD duration: PEA vs. CTRL, NS 2. AEs measurement: PEA vs. CTRL, NS
Marini et al., 2012 (Italy)	To assess PEA effect on pain in TMJ OA patients	PEA effect on joints, back, limbs, and chronic widespread pain and altered function	24 (PEA: 12; CTRL: 12)	1. Somatic measurement (Maximum active mouth opening, VAS) 2. AEs measurement (Self-administered questionnaire)	1. Somatic measurement: (a) **ΔVAS (day 14 -baseline): PEA > CTRL**; (b) ΔMaximum **Mouth Opening (day 14 -baseline): PEA > CTRL** 2. AEs measurement: PEA vs. CTRL, NS
Masek et al., 1974 (Czech Republic)	To assess PEA effect on the incidence and severity of URTI	PEA effect on respiratory tract infections and allergy symptoms	1. First trial: 468 (PEA: 223; PLB: 221) 2. Second trial: 918	1. Somatic measurement (Clinical assessment) 2. QoL measurement (Disability assessment)	First trial: **Episodes of fever/headache/sore throat, Nasal stuffiness/discharge/cough: PEA < PLB**; **Total number of patients: PEA <. PLB (6 weeks)**; PEA vs. PLB, NS (8 weeks); **Total number of days of illness: PEA < PLB (6 weeks, 8 weeks)** Second trial: **Out-patients, In-patients, Total number of patients: PEA < PLB (6 weeks, 8 weeks)**; Average duration of illness/fever: PEA vs. PLB, NS
Murina et al., 2013 (Italy)	To assess PEA effect on vestibulodynia in TENS-treated patients	PEA effect on gynecological and genitourinary pain or altered function	20 (PEA: 10; PLB: 10)	1. Visceral measurement (VAS, Marinoff dyspareunia scale, CPT) 2. AEs measurement (Clinical assessment)	1. Visceral measurement: (a) Post-treatment VAS, (b) Post-treatment Marinoff dyspareunia scale: PEA vs. PLB, NS 2. AEs measurement: PEA vs. PLB, NS
Orefice et al., 2016 (Italy)	To assess PEA effect on IFN-β1a-induced AEs in RR-MS patients	1. PEA effect on attention, awareness, and cognition 2. PEA effect on mobility, spasticity, and sensory-motor deficits 3. PEA effect on cutaneous pain, erythema, and eczema 4. PEA effect on blood/plasma or other tissue biomarkers alterations	29 (PEA: 15; PLB: 14)	1. Somatic measurement (VAS, erythema width) 2. QoL measurement (MSQoL-54) 3. Blood/plasma and/or other tissue measurement (Inflammatory markers quantification, eCBs/NAEs quantification) 4. Neurological measurement (EDSS) 5. Neuropsychiatric measurement (PASAT)	1. Somatic measurement: (a) **VAS**: PEA vs. PLB, NS (baseline); **PEA < PLB (**month **6, month 12)**; (b) Erythema width: PEA vs. PLB, NS (baseline, month 6, month 12) 2. QoL measurement: (a) **Cognitive function: PEA > PLB (6 months, 12 months)**; (b) **Change in health: PEA > PLB (6 months)**; (c) other MSQoL domains: PEA vs. PLB, NS 3. Blood/plasma measurement: (a) **IFN-γ**, (b) **IL-17 serum levels**: PEA vs. PLB, NS (month 1); **PEA < PLB (month 3, month 6, month 12)**; (d) **PEA**, (e) **AEA plasma levels**: PEA vs. PLB, NS (month 1); **PEA > PLB (**month **3, month 6, month 9, month 12)**; (g) ΔCT FAAH, (h) ΔCT NAAA: PEA vs. PLB, NS; (h) **FAAH mRNA expression (relative to month 1)**: PEA vs. PLB, NS (month 3, month 6, month 9); **PEA < PLB (month 12)**; (j) Correlations between PEA serum levels and other eCBs/NAEs serum levels: **↑ PEA, ↑ OEA, ↑ AEA (month 3, month 6, month 9, month 12)**; (k) Correlations between PEA serum levels and cytokines serum levels: **↑ PEA, ↓ IFN-γ (month 3, month 6, month 12)**; **↑ PEA, ↓ IL-17, ↓ TNF-α (month 3, month 6, month 12)** 4. Neurological measurement: PEA vs. PLB, NS (month 1, month 3, month 6, month 9, month 12) 5. Neuropsychiatric measurement: PEA vs. PLB, NS (month 1, month 6, month 12)
Ottaviani et al., 2019 (Italy)	To assess PEA effect on neuropathic pain in BMS patients	PEA effect on neuropathic pain	35 (PEA: 18; PLB: 17)	1. Neurological measurement (NRS Scoring system) 2. AEs measurement (Clinical assessment)	1. Neurological measurement (Burning intensity over time): PEA vs. PLB, NS (baseline, 30 days); **PEA < PLB (60 days)**; PEA < PLB, NS (4 months after discontinuation) 2. AEs measurement: PEA vs. PLB, NS
Palma et al., 2016 (Italy)	To assess PEA effect on pulmonary function decline in ALS patients	PEA effect on altered muscle force and respiratory capacity	64 (PEA: 28; CTRL: 36)	Neurological measurement (FVC%, MRC scale, ulnar and phrenic nerve, CMAP amplitudes, ALSFRS-R scale)	**FVC%: baseline > 12 weeks > 24 weeks (CTRL)**; baseline vs. 12 weeks vs. 24 weeks, NS (PEA); MRC, CMAP amplitude, ALSFRS-R total: baseline vs. 12 weeks vs. 24 weeks, NS (both groups); **ALSFRS-R bulbar, ALSFRS-R respiratory: time x treatment effect (PEA)**; time x treatment effect (CTRL), NS; **Proportion of survivors (24 weeks): PEA > CTRL**
Pickering et al., 2022 (Australia)	To assess PEA effect on PNP, sleep quality, and mood changes in diabetic patients	1. PEA effect on mood, anxiety, and stress 2. PEA effect on sleep 3. PEA effect on neuropathic pain 4. PEA effect on blood/plasma or other tissue biomarkers alterations	70 (PEA: 35; PLB: 35)	1. Neurological measurement (BPI-DPN, NPSI) 2. Neuropsychiatric measurement (Sleep quality assessment, MOS-Sleep; psychological assessment, DASS-21) 3. Blood/plasma and/or other tissue measurement (HbA1c, FBG, inflammatory markers quantification) 4. AEs measurement (Clinical assessment) 5. QoL measurement (BPI-DPN, NPSI, MOS)	1. Neurological measurement: (a) **effect on pain severity (BPI-PN): time, ↓**; **treatment, ↓**; (b) **pain severity (BPI-PN)**: PEA vs. PLB, NS (baseline); **PEA < PLB (week 2, week 4, week 8)**; (c) **pain interference (BPI-PN)**: PEA vs. PLB, NS (baseline); **PEA < PLB (week 4, week 8)**; (d) **total pain score (NPSI)**: PEA vs. PLB, NS (baseline); **PEA < PLB (week 4, week 8)**; (e) **superficial pain (NPSI)**: PEA vs. PLB, NS (baseline); **PEA < PLB (week 4, week 8)**; (f) **deep pain (NPSI)**: PEA vs. PLB, NS (baseline); **PEA < PLB (week 4, week 8)**; (g) **paroxysmal pain (NPSI)**: PEA vs. PLB, NS (baseline); **PEA < PLB (week 4, week 8)**; (h) **evoked pain (NPSI)**: PEA vs. PLB, NS (baseline, week 4); PEA < PLB (week 8, trend effect); (i) **paresthesia (NPSI)**: PEA vs. PLB, NS (baseline); PEA **< PLB (week 4, week 8)**; (j) Influence of prescribed pain medications on treatment effect: NS 2. Neuropsychiatric measurement: (a**) sleep disturbance**: PEA vs. PLB, NS (baseline); PEA < PLB (week 4, trend effect); **PEA < PLB (week 8)**; (b) **sleep adequacy**: PEA vs. PLB, NS (baseline); **PEA > PLB (week 4, week 8)**; (c) sleep quantity: PEA vs. PLB, NS (baseline, week 4, week 8); (d) **daytime** somnolence: PEA vs. PLB, NS (baseline); **PEA < PLB (week 4, week 8)**; (e) snoring: PEA vs. PLB, NS (baseline, week 4, week 8); (f) **shortness of breath or headache**: PEA vs. PLB, NS (baseline, week 4); **PEA < PLB (week 8)**; (g) **sleep problem index**: PEA vs. PLB, NS (baseline); **PEA < PLB (week 4, week 8)**; (h) **depressive symptoms change**: **PEA > PLB (week 8)**; (i) stress levels change: PEA > PLB (week 8, trend effect); (j) anxiety symptoms change: PEA vs. PLB, NS (week 8) 3. Blood/plasma measurement: (a) FBG, (b) HbA1c mmol/L, (c) HbA1c %, (d) CRP, (e) fibrinogen: PEA vs. PLB, NS (baseline, week 8); (f) **CRP > 5.0 mg/L**, (g) **IL6**: PEA vs. PLB, NS (baseline); **PEA < PLB (week 8)** 4. AEs measurement: PEA vs. PLB, NS
Rao et al., 2021 (Australia)	To assess PEA effect on sleep quality and quantity in DSP patients	1. PEA effect on sleep 2. PEA effect on blood/plasma or other tissue biomarkers alterations	103 (PEA: 55; PLB: 48)	1. Neuropsychiatric measurement (PSQI, wrist actigraphy, consensus sleep diary, PROMIS sleep disturbance questionnaire, SIQ, ESS) 2. QoL measurement (SF-36) 3. Blood/plasma and/or other tissue measurement (Pathology markers quantification, safety markers quantification) 4. AEs measurement (Clinical assessment)	1. Neuropsychiatric measurement: (a) PSQI scores improvement: PEA vs. PLB, NS (all time-points); (b) Sleep actigraphy data: Sleep quality (1–5), Total sleep time (h), Sleep time (h), Interruptions (min), Sleep Continuity (1–5), Sleep percentage (%): PEA vs. PLB, NS (all time-points); (c) **Sleep diary data: Sleep onset latency (min)**: PEA vs. PLB, NS (baseline); **PEA < PLB (week 4, week 8)**; Sleep time (h), Interruptions (n), Interruptions (min): PEA vs. PLB, NS (all time-points); (d): Data from sleep questionnaires: PEA vs. PLB (all time-points) 2. QoL measurement (SF-36): PEA vs. PLB, NS 3. Blood/plasma and/or other tissue measurement: PEA vs. PLB, NS 4. AEs measurement (reported gastrointestinal issues): PEA vs. PLB, NS
Rao et al., 2023a (Australia)	To assess PEA effect on the incidence, duration, and severity of URTI	PEA effect on respiratory tract infections and allergy symptoms	426 (PEA: 213; PLB: 213)	1. Somatic measurement (URTI incidence, URTI duration, WURSS-24) 2. QoL measurement (SF-8) 3. AEs measurement (Clinical assessment)	1. Somatic measurement: (a) **incidence of URTI episodes**, (b) **number of participants sick at least once: PEA < PLB**; (c) duration of URTI episodes: PEA vs. PLB, NS; (d) **scratchy throat (WURSS-24)**, (e) **cough (WURSS-24): PEA < PLB**; (f) hoarseness (WURSS-24): PEA < PLB (trend effect); (g) breathe easily (WURSS-24): PEA > PLB (trend effect); (h) other symptoms (WURSS-24): PEA vs. PLB, NS; (i) number of people reporting symptoms (WURSS-24): PEA vs. PLB, NS 2. QoL measurement: all comparisons, NS 3. AEs measurement: PEA vs. PLB, NS
Rao et al., 2023b (Australia)	To assess PEA effect on redness, dry skin, scaling, and itchiness in atopic eczema patients	PEA effect on cutaneous pain, erythema, and eczema	72 (PEA: 36; CTRL: 36)	1. Somatic measurement (SA-EASI, NRS, POEM, use of topical anti-inflammatory creams) 2. QoL measurement (DLQI) 3. AEs measurement (Clinical assessment)	1. Somatic measurement: (a) **redness (SA-EASI)**, (b) **dryness (SA-EASI)**, (c) **Total POEM outcome score: Week 4 < Week 2 < baseline (PEA, CTRL)**; **PEA < CTRL (Week 4)**; other comparisons, NS; (d) **POEM severity scoring: Week 4 < Week 2 < baseline (PEA, CTRL)**; (e) **thickness (SA-EASI)**, (f) **scratches (SA-EASI)**, (g) **itchiness (SA-EASI): Week 4,** Week **2 < baseline (CTRL)**; **Week 4 < Week 2 < baseline (PEA)**; PEA vs. CTRL, NS 2. QoL measurement (DLQI): PEA vs. CTRL, NS (all comparisons) 3. AEs measurement: PEA vs. CTRL, NS
Rossi et al., 2020 (Italy)	To assess PEA effect on RGCs function, IOP, visual field, and QoL in POAG or NTG patients	PEA effect on intraocular pressure and visual field	42 (Starting-with-PEA group: 21; Switching-to-PEA group: 19)	1. Visceral measurement (PERG, visual field examination, IOP assessment) 2. QoL measurement (NEI-VFQ 25) 3. AEs measurement (Clinical assessment)	1. Visceral measurement: (a) **IOP values:** starting-with-PEA group vs. switching-to-PEA group, NS (baseline); **starting-with-PEA group < switching-to-PEA group** (**month 4)**; **starting-with-PEA group > switching-to-PEA group (month 8)**; (b) Visual field (MD, PSD): starting-with-PEA group vs. switching-to-PEA group, NS (baseline, month 4, month 8); (c) **PERG (P50-wave amplitude)**: starting-with-PEA group vs. switching-to-PEA group, NS (baseline); **starting-with-PEA group > switching-to-PEA group (month 4)**; **starting-with-PEA group < switching-to-PEA group (month 8)**; PERG (other measures): starting-with-PEA group vs. switching-to-PEA group, NS (baseline, month 4, month 8) 2. QoL measurement (**NEI-VFQ 25 Total mean**): starting-with-PEA group vs. switching-to-PEA group, NS (baseline); **starting-with-PEA group > switching-to-PEA group (month 4)**; **starting-with-PEA group < switching-to-PEA group (month 8)** 3. AEs measurement: starting-with-PEA group vs. switching-to-PEA group, NS
Salaffi et al., 2023 (Italy)	To assess PEA effect on chronic pain and general wellbeing in FM patients	PEA effect on joints, back, limbs, and chronic widespread pain and altered function	130 (PEA: 62; CTRL: 68)	1. Somatic measurement (WPI) 2. QoL measurement (FIQR, FASmod) 3. AEs measurement (Clinical assessment)	1. Somatic measurement (**WPI, AUC**): **PEA < CTRL** 2. QoL measurement: (a) **FIQR (AUC)**, (b) **FASmod (AUC): PEA < CTRL** 3. AEs measurement: PEA vs. CTRL, NS
Salehi et al., 2022 (Iran)	To assess PEA effect on negative symptoms in SCZ patients	1. PEA effect on mood, anxiety, and stress 2. PEA effect on psychotic symptoms and autistic behaviors	60 (PEA: 30; PLB: 30)	1. Neuropsychiatric measurement (PANSS, HDRS) 2. AEs measurement (ESRS, open-ended questions, comprehensive side effect checklist)	1. Neuropsychiatric measurement: (a) **Effect on PANSS negative: time, ↓**; **time × treatment interaction, ↓**; (b) **Effect on PANSS positive: time, ↓**; time × treatment interaction, NS; (c) **Effect on PANSS general: time, ↓**; **time × treatment interaction, ↓**; (d) **Effect on PANSS total: time, ↓**; **time × treatment interaction, ↓**; (e) Effect on HDRS: time × treatment interaction, NS 2. AEs measurement: (a) Effect on ESRS global score: time, NS; time × treatment interaction, NS; (b) Frequency of adverse events (drowsiness, dizziness, tremor, increased appetite, nervousness, restlessness, skin rash, blurred vision, fatigue, diarrhea, dry mouth, sore throat, tachycardia): PEA vs. PLB, NS
Steels et al., 2019 (Australia)	To assess PEA effect on pain in knee OA patients	1. PEA effect on mood, anxiety, and stress 2. PEA effect on sleep 3. PEA effect on joints, back, limbs, and chronic widespread pain and altered function 4. PEA effect on blood/plasma or other tissue biomarkers alterations	111 (PEA: 71; PLB: 40)	1. Somatic measurement (WOMAC; NRS; use of rescue medication) 2. Neuropsychiatric measurement (DASS, PSS, PSQI) 3. QoL measurement (SF-36, WOMAC function subdomain) 4. AEs measurement (Clinical assessment) 5. Blood/plasma and/or other tissue measurement	1. Somatic measurement: (a) **WOMAC total score:** PEA(300 mg) vs. PEA(600 mg) vs. PLB, NS (baseline, week 4); **PEA(300 mg), PEA(600 mg) < PLB (week 8)**; (b) **WOMAC pain score:** PEA(300 mg) vs. PEA(600 mg) vs. PLB, NS (baseline, week 1, week 4); **PEA(300 mg), PEA(600 mg) < PLB (week 8)**; (c) **WOMAC stiffness score:** PEA(300 mg) vs. PEA(600 mg) vs. PLB, NS (baseline, week 4); **PEA(300 mg), PEA(600 mg) < PLB (week 8)**; (d) **WOMAC function score:** PEA(300 mg) vs. PEA(600 mg) vs. PLB, NS (baseline, week 4); PEA(300 mg) vs. PLB, NS (week 8); **PEA(600 mg) < PLB (week 8)**; (e) **NRS worst pain yesterday:** PEA(300 mg) vs. PEA(600 mg) vs. PLB, NS (baseline, week 4); **PEA(300 mg), PEA(600 mg) < PLB (week 8)**; (f) **NRS least pain yesterday:** PEA(300 mg) vs. PEA(600 mg) vs. PLB, NS (baseline, week 4); **PEA(300 mg), PEA(600 mg) < PLB (week 8)**; (g) **NRS average pain**: PEA(300 mg) vs. PEA(600 mg) vs. PLB, NS (baseline); **PEA(300 mg), PEA(600 mg) < PLB (week 1, week 4, week 8)** 2. Neuropsychiatric measurement: (a) DASS anxiety score: PEA(300 mg) vs. PEA(600 mg) vs. PLB, NS (baseline); PEA(300 mg), PEA(600 mg) < PLB (week 8); (b) DASS depression score: PEA(300 mg) vs. PEA(600 mg) vs. PLB, NS (baseline, week 8); (c) PSI stress score: PEA(300 mg) vs. PEA(600 mg) vs. PLB, NS (baseline, week 8); (d) sleep quality (PSQI, SF-36): PEA(300 mg) vs. PEA(600 mg) vs. PLB, NS (baseline, week 8) 3. Blood/plasma and/or other tissue measurement (Biochemical, hematological, and inflammatory parameters): PEA(300 mg) vs. PEA(600 mg) vs. PLB, NS (baseline, week 8) 4. AEs measurement: PEA(300 mg) vs. PEA(600 mg) vs. PLB, NS (baseline, week 8)
Strobbe et al., 2013 (Italy)	To assess PEA effect on systemic endothelial function in OH patients	PEA effect on intraocular pressure and visual field	80 (PEA: 40; PLB: 40)	1. Visceral measurement (Brachial artery FMD, IOP) 2. AEs measurement (Clinical assessment)	1. Visceral measurement: (a) **FMD values: T1, T2, T3 > T0 (starting-with-PEA group)**; other comparisons, NS (starting-with-PEA group); **T3 > T0, T1, T2**; other comparisons, NS (switching-to-PEA group); (b) **IOP values: T1, T2, T3 < T0 (starting-with-PEA group)**; other comparisons, NS (starting-with-PEA group); **T3 < T0, T1, T2 (switching-to-PEA group)**; other comparisons, NS (switching-to-PEA group) 2. AEs measurement: starting-with-PEA group vs. switching-to-PEA group, NS
Tartaglia et al., 2015 (Italy)	To assess PEA effect on pelvic pain in primary dysmenorrhea patients	PEA effect on gynecological and genitourinary pain or altered function	220 (PEA: 110; PLB: 110)	1. Visceral measurement (Questionnaire, 10-point VAS) 2. AEs measurement (Clinical assessment)	1. Visceral measurement (VAS score): (a) **improvement of pelvic pain: PEA > PLB**; (b) **mean improvement of pelvic pain: PEA > PLB** 2. AEs measurement: PEA vs. PLB, NS
Versace et al., 2023 (Italy)	To assess PEA effect over reduced LICI in patients with post-COVID-19 cognitive dyfunction and fatigue	PEA effect on attention, awareness, and cognition	39 (PEA: 17; PLB: 17)	Neuropsychiatric measurement (LICI protocol, SAI, LTP-like cortical plasticity, MoCA, FAB)	**Effect on % change of test MEP (LICI): time × treatment interaction**; **% of test MEP (LICI): PEA group: POST < PRE**; PLB group: POST vs. PRE, NS; Effect on % change of test MEP (SAI): time × treatment interaction, NS; % of test MEP (SAI): POST vs. PRE, NS (PEA group, PLB group); **Effect on MEP amplitude (LTP-like cortical plasticity): time × treatment interaction (1 and 10 min following iTBS)**; time × treatment interaction, NS (20 min following iTBS); **Mean amplitude of MEPs (LTP-like cortical plasticity): PEA group: POST > PRE (1 and 10 min following iTBS)**; POST vs. PRE, NS (20 min following iTBS); PLB group: all comparisons, NS RMT protocol: all comparisons, NS Effect on cognitive performance and executive functions (MoCA, FAB): treatment × time interaction, NS
Yuan et al., 2014 (China)	To assess PEA effect on dry skin and itchy sensation in asteatotic eczema patients	PEA effect on cutaneous pain, erythema, and eczema	66 (PEA: 30; CTRL: 30)	1. Somatic measurement (EASI, skin hydration, TEWL, CPT) 2. AEs measurement (Clinical assessment)	1. Somatic measurement: (a) Symptom improvement: **skin scaling**, dryness, **itching**: **PEA > CTRL (28 days)**; other symptoms: PEA vs. CTRL, NS; (b) Skin barrier function: **ΔCapacitance: PEA > CTRL (3, 7, 14, 28 days)**; ΔTEWL: PEA vs. CTRL, NS (all time-points); (c) **CPT**: **PEA > CTRL (14 days)**; other time-points: PEA vs. CTRL, NS 2. AEs measurement: PEA vs. CTRL, NS

↑, Increased; ↓, Decreased; <, less/smaller than; >, more/greater than; ≥, more/greater than or equal to; 2-AG, 2-Arachidonoylglycerol; ABC-C, Aberrant Behavior Checklist-Community; AEA, Anandamide; AEs, Adverse Events; AIS, Acute Ischemic Stroke; ALS, Amyotrophic Lateral Sclerosis; ALA, Alpha-lipoic acid; ALSFRS-R, ALS Functional Rating Scale–Revised; AR, Allergic Rhinitis; ASD, Autism Spectrum Disorder; AUC, Area Under the Curve; BCTQ, Boston Carpal Tunnel Questionnaire; BCVA, Best-corrected visual acuity; BDI, Beck Depression Inventory; BMS, Burning mouth syndrome; BNCE, Brief Neuropsychological Cognitive Examination; BOP, Bleeding on probing; BPAD, Bipolar Affective Disorder; BPFS, Back Pain Functional Scale; BPI-DPN, Brief Pain Inventory-Diabetic Peripheral Neuropathy; BSS, Bristol Stool Scale; C/D, Cup-to-disc; CA-125, Cancer Antigen 125; CAL, Clinical attachment level; CB1, Cannabinoid receptor type 1; CB2, Cannabinoid receptor type 2; CCT, Central corneal thickness; CD3, Cluster of differentiation 3 lymphocytes; CD8, Cluster of differentiation 8 lymphocytes; CMAP, Compound Muscle Action Potential; COD, Chronic olfactory dysfunction; Co-ultraPEALut, Co-ultramicronized PEA plus Luteolin; COVID-19, SARS-CoV-2 infection; CP/CPPS, Chronic Prostatitis/Chronic Pelvic Pain syndrome; CPSS, Cincinnati Prehospital Stroke Scale; CPT, Current perception threshold; CRP, C-Reactive Protein; CTRL, Control; CTS, Carpal Tunnel Syndrome; DASS, Depression Anxiety and Stress Scale; DLQI, Dermatology Quality of Life Index; DOM, Delirium-O-Meter; DSP, Disturbed Sleeping Pattern; EASI, Eczema Area and Severity Index; eCBs/NAEs, Endocannabinoids/N-Acylethanolamines; EDSS, Expanded Disability Status Scale; EIA, Enzyme immunoassay; ELISA, Enzyme-linked immunosorbent assay; EMS, Endometriosis; ENG, Electroneurography; ESR, Erythrocyte Sedimentation Rate; ESRS, Extrapyramidal Symptom Rating Scale; ESS, Epworth Sleepiness Scale; FAAH, Fatty Acid Amide Hydrolase; FAB, Frontal Assessment Battery; FASmod, FBG, Fasting Blood Glucose; FIQR, Fibromyalgia Impact Questionnaire; FM, Fibromyalgia; FMD, Flow-mediated vasodilation; FORD, Free Oxygen Radical Defense; FORT, Free Oxygen Radical Test; FSS, Functional Status Scale; FVC, Forced Vital Capacity; GAD-10, Generalized Anxiety Disorder Assessment; GCF, Gingival crevicular fluid; GCS, Glasgow Coma Scale; GFR, Glomerular Filtration Rate; GR, Gingival recessions; h, hours; HbA1c, Glycated Haemoglobin; HC, Healthy controls; HDRS, Hamilton Depression Rating Scale; IBS, Irritable Bowel Syndrome; IBS-C, IBS with predominant constipation; IBS-D, IBS with predominant diarrhea; IBS-M, IBS with mixed symptoms; IBS-SSS, IBS-Severity Scoring System; IFN-β1a, Interferon-Beta1a; IFN-γ, Interferon-Gamma; IgG, Immunoglobulin G; IIEF5, International Index of Erectile Function 5; IL-4, Interleukin-4; IL-8, Interleukin-8; IL-10, Interleukin-10; IL-17, Interleukin-17; IL-1β, Interleukin-1Beta; IL-6, Interleukin-6; ILTM, Impacted lower third molar; IOP, Intra-ocular pressure; IPSS, International Prostatic Symptoms Score; ISI, Insomnia Severity Index; iTBS, Intermittent theta burst stimulation; ITT, Intention-to-treat; LC-APCI-MS, Liquid chromatography-atmospheric pressure chemical-ionization-mass spectrometry; LICI, Long-interval intracortical inhibition; LTP, Long-term potentiation; LWT, Last Week of Treatment; MC, Mast cells; MCV, Mean Corpuscular Volume; MD, Mean defect; MDD, Major Depressive Disorder; MDI, Major Depression Inventory; MDQ, Mood Disorder Questionnaire; MEP, Motor evoked potential; min, minutes; MMSE, Mini Mental State Examination; MoCA, Montreal Cognitive Assessment; MOS, Medical Outcome Study; MRC, Medical Research Council; mRNA, Messenger RNA; mRS, Modified Rankin Scale; MSQoL-54, Multiple Sclerosis Quality of Life-54; MTS, Modified Tardieu Scale; NAAA, N-Acylethanolamine Acid Amidase; NEI-VFQ 25, 25 item National Eye Institute Visual Function Questionnaire; NGF, Nerve Growth Factor; NIH/CPSI, National Institutes of Health Chronic Prostatitis Symptom Index; NIHSS, National Institutes of Health Stroke Scale; NNT, Number Needed to Treat; NPSI, Neuropathic Pain Symptom Inventory; NRS, Numeric Rating Scale; NS, Not Significant; NSAIDs, Nonsteroidal anti-inflammatory drugs; NTG, Normal Tension Glaucoma; OA, Osteoarthritis; OEA, Oleoylethanolamide; OH, Ocular hypertension; OT, Olfactory Training; PANSS, Positive and Negative Syndrome Scale; PASAT, Paced Auditory Serial Addition Test; PD, Probing depth; PEA, Palmitoylethanolamide; PERG, Pattern electroretinogram; PGIC, Patient Global Impression of Change; PI, Plaque score; PLB, Placebo; PLT, Platelet; PNP, Peripheral neuropathy; POAG, Primary open glaucoma; POD, Post-operative delirium; POEM, Patient-Oriented Eczema Measure; PP, Per protocol; PROMIS, Patient Reported Outcomes Measurement Information System; PSD, Pattern standard deviation; PSQI, Pittsburgh Sleep Quality Index; PSS, Perceived Stress Scale; PT, Prothrombin Time; QoL, Quality of Life; RDQ, Rolant-Morris disability questionnaire; RGCs, Retinal ganglion cells; RR-MS, Relapsing-Remitting Multiple Sclerosis; rTNSS, Reflective Total Nasal Symptom Score; S-TOPS, Pain Survey; SA-EASI, Self-Assessed Eczema Area and Severity Index; SAI, Short-latency sensory afferent inhibition; SAP, Sensory action potentials; SCI, Spinal Cord Injury; SCZ, Schizophrenia; SF-8, 8-item Health Survey; SF-36, 36-item Health Survey; SIQ, The Sleep Inertia Questionnaire; SNCV, Sensory nerve conduction velocity; SSS, Symptom Severity Scale; TBI, Traumatic Brain Injury; TDI, Threshold, Discrimination and Identification subtests; TENS, Transcutaneous electrical nerve stimulation; TEWL, Transepidermal water loss; TMJ, Temporo-mandibular joint; Tn, Timepoint; TNF-α, Tumor Necrosis Factor-Alpha; URTI, Upper Respiratory Tract Infections; US, Ultrasound; VAS, Visual Analogue Scale; VNS, Visual Numeric Scale; vs., compared to; WOMAC, Western Ontario and McMaster Universities Arthritis Index; WPI, Widespread Pain Index; WURSS-24, Wisconsin Upper Respiratory Symptom Survey; YMRS, Young Mania Rating Scale; Δ, Difference between values; ΔCT, The Comparative Ct Method. Bold font emphasizes statistically significant results.

Additional data on methodological quality of studies are reported in Table 2.

**Table 2:** Methodological characteristics of Randomized Controlled Trials investigating Palmitoylethanolamide supplementation across human diseases.

Study ID	Level of concealment	Defined population	Age (years, mean ± SD)	Gender (count, %)	PEA measure (dosage, formulation, intake route)	PEA adequate evaluation (period of exposure, use as monotherapy or add-on)
Abedini et al., 2022 (Iran)	Double-blind	Bipolar affective disorder with acute mania (SCID-5, MINI)	PEA: 30.8 ± 9.8	PEA: Female 9 (28.1%)/Male 23 (71.9%)	Undeclared PEA formulation (600 mg/bid, capsule, oral)	6 weeks, add-on to lth and rsp
			PLB: 32.7 ± 9.0	PLB: Female 11 (35.5%)/Male 20 (64.5%)		
Albanese et al., 2022 (Italy)	X	COVID-19 (positive nasopharyngeal swab for SARS-CoV-2)	PEA: 45.6 ± 13.7	PEA: Female 28 (62.2%)/Male 17 (37.8%) CTRL: Female 23 (51.1%)/Male 22 (48.9%)	mPEA + umPEA (900 mg/bid, microgranules, sublingual)	4 weeks, add-on to TAU
			CTRL: 55.8 ± 22.5			
Andresen et al., 2016 (Denmark, Norway, United Kingdom)	Double-blind	Neuropathic pain following spinal cord injury (SCI-compatible pain distribution, MRI)	PEA: 58.6 ± 11.3	PEA: Female 14 (39.0%)/Male 22 (61.0%)	umPEA (600 mg/bid, microgranules, sublingual)	12 weeks, add-on to TAU
			PLB: 54.1 ± 11.7	PLB: Female 5 (14.0%)/Male 32 (86.0%)		
Bacci et al., 2011 (Italy)	Single-blind	Impacted lower third molars (X-Rays, Pell and Gregory classification)	X	X	mPEA (300 mg/bid, tablet, oral)	2 weeks, monotherapy (6 days before surgery, 9 days afterwards)
Bonzanino et al., 2024 (Italy)	Open-label	Acute ischemic stroke (NIHSS)	Mean age of the sample: 77.9 ± 10.3	Female 27 (45.0%)/Male 33 (55.0%)	Co-ultraPEALut (umPEA 700 mg + luteolin 70 mg/bid, suspension, oral)	12 weeks, add-on to TAU (starting within 72 h after ischemic event)
Briskey et al., 2023 (Australia)	Double-blind	Allergic rhinitis (rTNSS)	PEA: 45.6 ± 15.6	PEA: Female 34 (65.4%)/Male 18 (34.6%)	LipiSperse PEA (300 mg/day, capsule, oral)	2 weeks, monotherapy
			PLB: 45.3 ± 12.8	PLB: Female 38 (77.6%)/Male 11 (22.4%)		
Briskey et al., 2024 (Australia)	Double-blind	Migraine (ICHD3)	PEA: 41.2 ± 11.5	PEA: Female 35 (87.5%)/Male 5 (12.5%)	LipiSperse PEA (300 mg/bid, capsule, oral)	8 h (one dose upon commencement of migraine symptoms, a second dose if migraine had not resolved 2 h post-dose), monotherapy
			PLB: 43.8 ± 10.8	PLB: Female 35 (87.5%)/Male 5 (12.5%)		
Campolo et al., 2021 (Italy)	Single-blind	Traumatic Brain Injury (GCS)	PEA: 49.7 ± 17.9	PEA: Female 2 (13.3%)/Male 13 (86.7%)	Co-ultraPEALut (umPEA 700 mg + luteolin 70 mg/bid, tablet, oral)	26 weeks, add-on to TAU
			CTRL: 53.8 ± 17.5	CTRL: Female 4 (26.7%)/Male 11 (73.3%)		
Cantone et al., 2024 (Italy)	Double-blind	Post-COVID-19 anosmia/hyposmia and parosmia (Positive nasopharyngeal swab for SARS-CoV-2)	Co-ultraPEALut: 44.8 ± 12.2	Co-ultraPEALut: Female 11 (64.7%)/Male 6 (35.3%)	Co-ultraPEALut (umPEA 700 mg + luteolin 70 mg/day, sachet, oral) Co-ultraPEALut/ALA (umPEA 700 mg + luteolin 70 mg + ALA 600 mg/day, sachet, oral)	12 weeks, monotherapy (after OT)
			Co-ultraPEALut/ALA: 42 ± 10.3	Co-ultraPEALut/ALA: Female 18 (64.3%)/Male 10 (35.7%)		
			ALA: 35.9 ± 12.3	ALA: Female 9 (42.9%)/Male 12 (57.1%)		
			PLB: 52.1 ± 11.8	PLB: Female 13 (56.5%)/Male 10 (43.5)		
Cobellis et al., 2011 (Italy)	Double-blind	Endometriosis (ESHRE, laparoscopic conservative surgery, histological examination)	PEA: 26–37	N/A	mPEA/transpolydatin (mPEA 400 mg + transpolydatin 40 mg/bid, tablet, oral)	12 weeks, monotherapy
			PLB: 25–41			
			Celecoxib: 24–35			
Coraci et al., 2018 (Italy)	Double-blind	Minimum/mild idiopathic Carpal tunnel syndrome (Padua's classification)	X	X	Undeclared PEA formulation (600 mg/day, tablet, oral)	4 weeks, monotherapy
Costagliola et al., 2014 (Italy)	Open-label	Normal tension glaucoma (Clinical assessment, IOP, BCVA, visual field test, GHT)	Mean age of the sample: 53.7 (31–67)	PEA: Female 10 (62.5%)/Male 6 (37.5%) CTRL: Female 10 (62.5%)/Male 6 (37.5%)	umPEA (300 mg/bid, tablet, oral)	24 weeks, monotherapy
Cremon et al., 2017 (Italy, Spain, France, Croatia, Bosnia)	Double-blind	Irritable bowel syndrome (Clinical assessment)	PEA: 37.0 ± 10.8	PEA: Female 18 (62.1%)/Male 11 (37.9%)	mPEA/polydatin (mPEA 200 mg + polydatin 20 mg/day, tablet, oral)	12 weeks, monotherapy
			PLB: 40.4 ± 9.8	PLB: Female 11 (44.0%)/Male 14 (56.0%)		
D'Ascanio et al., 2021 (Italy)	Single-blind	Post-COVID-19 anosmia/hyposmia (Positive nasopharyngeal swab for SARS-CoV-2, Sniffin’ Sticks)	42.2 ± 14.1	PEA: Female 5 (71.4%)/Male 2 (28.6%)	Co-ultraPEALut (umPEA 700 mg + luteolin 70 mg/day, tablet, oral)	4 weeks, monotherapy (after OT)
				PLB: Female 3 (60.0%)/Male 2 (40.0%)		
Di Nardo et al., 2024 (Italy)	Double-blind	Irritable bowel syndrome (Rome IV criteria)	PEA: 13.7 ± 2.3	PEA: Female 18 (52.9%)/Male 16 (47.1%)	mPEA/polydatin (mPEA 200 mg + polydatin 20 mg/tid, tablet, oral)	12 weeks, monotherapy
			PLB: 13.8 ± 2.1	PLB: Female 20 (55.6%)/Male 16 (44.4%)		
Di Stadio et al., 2022 (Italy)	Double-blind	Post-COVID-19 COD (Positive nasopharyngeal swab for SARS-CoV-2, Sniffin’ Sticks)	PEA: 42.1 ± 14.5	PEA: Female 83 (63.8%)/Male 47 (36.2%)	Co-ultraPEALut (umPEA 700 mg + luteolin 70 mg/day, tablet, oral)	12 weeks, monotherapy (prior to OT)
			PLB: 47.0 ± 14.6	PLB: Female 38 (69.0%)/Male 17 (31.0%)		
Di Stadio et al., 2023a (Italy)	Double-blind	Post-COVID-19 anosmia/parosmia (Positive nasopharyngeal swab for SARS-CoV-2, 16-pen version of the Sniffin’ Sticks)	PEA: 36.7 ± 11.8	PEA: Female 49 (52%)/Male 45 (48%)	Co-ultraPEALut (umPEA 700 mg + luteolin 70 mg/day, tablet, oral)	12 weeks, monotherapy (prior to OT)
			PLB: 50.5 ± 12.7	PLB: Female 21 (58%)/Male 15 (42%)		
Di Stadio et al., 2023b (Italy)	Double-blind	Post-COVID-19 anosmia/hyposmia (Positive nasopharyngeal swab for SARS-CoV-2, Sniffin’ Sticks)	PEAdaily: 39.8 ± 11.5	PEAdaily: Female 18 (37.5%)/Male 30 (62.5%)	Co-ultraPEALut (umPEA 700 mg + luteolin 70 mg/day or/bid, sachet, oral)	12 weeks, monotherapy or add-on to OT
			PEAbid: 37.1 ± 13.9	PEAbid: Female 24 (60.0%)/Male 16 (40.0%)		
			PEA + OT: 42.5 ± 13.5	PEA + OT: Female 40 (71.4%)/Male 16 (28.6%)		
			PLB + OT: 40.9 ± 11.7	PLB + OT: Female 26 (68.4%)/Male 12 (31.6%)		
Evangelista et al., 2018 (Italy)	Open-label	Carpal Tunnel Syndrome (Clinical Practice Guidelines, AAOS)	PEA: 57.4 ± 11.5	PEA: Female 17 (77.3%)/Male 5 (22.7%)	1. umPEA (600 mg/bid, microgranules, sublingual) 2. umPEA (600 mg/day, tablet, oral)	1. 60 days prior to surgery: (a) 10 days (sublingual), monotherapy; (b) 50 days (oral), monotherapy 2. After surgery: (a) 60 days (oral), monotherapy; (b) 30 days (oral), monotherapy
			CTRL: 56.7 ± 17.2	CTRL: Female 11 (55.0%)/Male 9 (45.0%)		
Faig-Marti and Martinez-Catassus, 2017 (Spain) Faig-Marti and Martinez-Catassus, 2020 (Spain)	Double-blind	Mild/moderate Carpal Tunnel Syndrome (ENG)	PEA: 51.8 ± 11.1	PEA: Female 18 (60.0%)/Male 12 (40.0%)	Undeclared PEA formulation (600 mg/day, tablet, oral)	8 weeks, monotherapy
			PLB: 53.3 ± 13.4	PLB: Female 19 (61.3%)/Male 12 (38.7%)		
Gagliano et al., 2011 (Italy)	Double-blind	Primary open angle glaucoma/Ocular hypertension (IOP, Clinical assessment)	Group A: 65.0 ± 12.0	Group A: Female 17 (81.0%)/Male 4 (19.0%)	Undeclared PEA formulation (600 mg/day, tablet, oral)	Group A: 8 weeks, monotherapy, before 4-week washout and 8-week PLB Group B: 8 weeks, monotherapy, after 4-week washout and 8-week PLB
			Group B: 63.0 ± 11.0	Group B: Female 14 (66.7%)/Male 7 (33.3%)		
Germini et al., 2017 (Italy)	Double-blind (N-of-1 trial)	Chronic non-cancer non-ischemic pain in the back, joints, or limbs (Clinical assessment)	PEA: 83.2 ± 4.6	PEA: Female 5 (100%)	umPEA (600 mg/bid, tablet, oral)	3 weeks, monotherapy (prior to/after a 2-week wash-out period)
			PLB: 83.2 ± 4.6	PLB: Female 5 (100%)		
Ghazizadeh-Hashemi et al., 2018 (Iran)	Double-blind	Major Depressive Disorder (SCID-5)	PEA: 35.4 ± 7.1	PEA: Female 8 (30.0%)/Male 19 (70.0%)	umPEA (600 mg/bid, capsule, oral)	6 weeks, add-on to ctp
			PLB: 33.9 ± 6.6	PLB: Female 11 (40.0%)/Male 16 (60.0%)		
Giammusso et al., 2017 (Italy)	Single-blind	Chronic prostatitis/chronic pelvic pain syndrome (IPSS, NIH-CPSI, PSA)	Mean age of the sample: 41.32 ± 1.686	X	PEA/ALA (PEA 300 mg + ALA 300 mg/day, capsule, oral)	12 weeks, monotherapy
Guida et al., 2010 (Italy)	Double-blind	Radicular and/or core compression of the sciatic nerve and discopathy (Clinical assessment)	PEA(300 mg): 42.0 ± 10.7	PEA(300 mg): Female 98 (46.2%)/Male 114 (53.8%)	mPEA (300 mg/day or 300 mg/bid, capsule, oral)	3 weeks, monotherapy
			PEA(600 mg): 43.0 ± 11.4	PEA(600 mg): Female 99 (46.0%)/Male 116 (54.0%)		
			PLB: 43.6 ± 11.5	PLB: Female 103 (49.3%)/Male 106 (50.7%)		
Isola et al., 2021 (Italy)	Double-blind	Periodontitis (Clinical assessment)	Mean age of the sample: 47.8	Total sample: Female 32 (48.5%)/Male 34 (51.5%)	PEA/baicalin (PEA 800 mg + baicalin 400 mg/bid, capsule, oral)	5 days, add-on to SRP
Kadanangode et al., 2023 (India)	Open-label	Arthritis (Clinical assessment, DAS 28)	PEA: 41.73 ± 9.85	X	PEA/astaxanthin (PEA 800 mg + astaxanthin 2 mg/day, tablet, oral)	12 weeks, add-on to TAU
			CTRL: 45.83 ± 6.68			
Khalaj et al., 2018 (Iran)	Double-blind	Autism Spectrum Disorder (DSM-5)	PEA: 6.84 ± 2.1	PEA: Female 9 (29.0%)/Male 22 (71.0%)	Undeclared PEA formulation (600 mg/bid, capsule, oral)	10 weeks, add-on to rsp
			PLB: 7.42 ± 2.35	PLB: Female 6 (19.4%)/Male 25 (80.6%)		
Lunardelli et al., 2019 (Italy)	Single-blind	Post-operative Delirium (DSM-5, CAM, 4AT test, DOM scale)	PEA: 87.2 ± 5.2	PEA: Female 33 (82.5%)/Male 7 (17.5%)	Co-ultraPEALut (umPEA 700 mg + luteolin 70 mg/bid, tablet, oral)	Up to 3 days after surgery, monotherapy (at 12-h intervals, not later than 12 h from admission)
			CTRL: 87.3 ± 5.6	CTRL: Female 37 (92.5%)/Male 3 (7.5%)		
Marini et al., 2012 (Italy)	Triple-blind	Temporo-mandibular disorder (RDC/TMD)	X	Total sample: Female 16 (66.7%)/Male 8 (33.3%)	Undeclared PEA formulation (300 mg + 600 mg/day, 300 mg/bid, tablet, oral)	2 weeks, monotherapy (900 mg/day for 7 days, 600 mg/day for 7 days)
Masek et al., 1974 (Czech Republic)	Double-blind	Upper respiratory tract infections (temperature ≥37.5 °C, headache, sore throat, myalgia, nasal stuffiness, or discharge, productive or dry cough, malaise, fatigue)	First trial: PEA: 37.4 ± 1.5 PLB: 35.4 ± 1.26; Second trial: X	First trial: PEA: Female 125 (66.0%)/Male 98 (34.0%) PLB: Female 136 (61.5%)/Male 85 (38.5%); Second trial: X	Impulsin (300 mg x2/tid, tablet, oral)	12 days, monotherapy
Murina et al., 2013 (Italy)	Double-blind	Vestibulodynia (≥6 months history of vulvar pain upon tampon insertion or attempted intercourse, positive cotton swab test result)	PEA: 34.4 (18-48) PLB: 31.8 (23-47)	N/A	PEA/transpolydatin (PEA 400 mg + transpolydatin 40 mg/bid, tablet, oral)	8 weeks, add-on to TENS
Orefice et al., 2016 (Italy)	Double-blind	RR-MS (McDonald's 2010 criteria)	PEA: 30.60 ± 7.60 PLB: 28.93 ± 4.86	PEA: Female 6 (40.0%)/Male 9 (60.0%) PLB: Female 9 (64.0%)/Male 5 (36.0%)	umPEA (600 mg/day, tablet, oral)	53 weeks, monotherapy
Ottaviani et al., 2019 (Italy)	Double-blind	Burning mouth syndrome (IHCC)	X	PEA: Female 13 (72.2%)/Male 5 (27.8%) PLB: Female 16 (94.1%)/Male 1 (5.9%)	umPEA (600 mg/bid, microgranules, sublingual)	8 weeks, monotherapy
Palma et al., 2016 (Italy)	Single-blind	Amyotrophic lateral sclerosis (El Escorial diagnostic criteria)	PEA: 65.3 ± 10.2	PEA: Female 12 (42.9%)/Male 16 (57.1%)	umPEA (600 mg/bid, tablet, oral)	24 weeks, add-on to rlz
			CTRL: 62.1 ± 9.5	CTRL: Female 17 (47.2%)/Male 19 (52.8%)		
Pickering et al., 2022 (Australia)	Quadruple-blind	Peripheral neuropathy in diabetic patients (Clinical assessment, S-LANSS, DN4)	PEA: 65.5 (53-79)	PEA: Female 13 (39.4%)/Male 20 (60.6%)	LipiSperse PEA (300 mg/bid, capsule, oral)	8 weeks, monotherapy
			PLB: 61.5 (32–75)	PLB: Female 18 (54.5%)/Male 15 (45.5%)		
Rao et al., 2021 (Australia)	Double-blind	Disturbed sleeping pattern (PSQI)	X	PEA: Female 30 (54.5%)/Male 25 (45.5%) PLB: Female 34 (70.8%)/Male 14 (29.2%)	LipiSperse PEA (150 mg/bid, capsule, oral)	8 weeks, monotherapy (1 h prior to sleep)
Rao et al., 2023a (Australia)	Double-blind	Upper respiratory tract infections (Clinical assessment)	PEA: 40.0 ± 12.5	PEA: Female 28 (52.8%)/Male 25 (47.2%)	LipiSperse PEA (300 mg/bid, capsule, oral)	12 weeks, monotherapy
			PLB: 38.9 ± 11.7	PLB: Female 29 (53.7%)/Male 25 (46.3%)		
Rao et al., 2023b (Australia)	Double-blind	Atopic dermatitis (Clinical assessment)	PEA: 37.4 ± 11.7	PEA: Female 27 (84.4%)/Male 5 (15.6%)	LipiSperse PEA (PEA 1.5% concentrated/bid, moisturizing cream, skin application)	4 weeks, monotherapy
			CTRL: 36.0 ± 10.6	CTRL: Female 21 (63.6%)/Male 12 (36.4%)		
Rossi et al., 2020 (Italy)	Single-blind	Primary open angle glaucoma/Normal tension glaucoma (Clinical assessment, IOP)	Starting-with-PEA group: 67.6 (62.3–75.5)	Starting-with-PEA group: Female 11 (52.4%)/Male 10 (47.6%)	umPEA (600 mg/day, tablet, oral)	16 weeks, add-on to current topical therapy
			Switching-to-PEA group: 66.7 (55.7–70.0)	Switching-to-PEA group: Female 8 (42.1%)/Male 11 (57.9%)		
Salaffi et al., 2023 (Italy)	Open-label	Fibromyalgia (ACR)	PEA: 52.9 ± 12.1	PEA: Female 62 (100.0%)/Male 0 (0.0%)	PEA/ALC (PEA 600 mg + ALC 500 mg/bid, tablet, oral)	12 weeks, add-on to dlx and pgb
			CTRL: 53.4 ± 13.12	PLB: Female 68 (100.0%)/Male 0 (0.0%)		
Salehi et al., 2022 (Iran)	Double-blind	Chronic Schizophrenia (SCID-5)	PEA: 33.8 ± 6.9	PEA: Female 7 (8.0%)/Male 23 (92.0%)	Undeclared PEA formulation (600 mg/bid, capsule, oral)	8 weeks, add-on to rsp
			PLB: 36.8 ± 9.6	PLB: Female 9 (16.0%)/Male 21 (84.0%)		
Steels et al., 2019 (Australia)	Double-blind	Knee osteoarthrits (Clinical assessment)	PEA(300 mg): 57.0 ± 11.0	PEA(300 mg): Female 20 (66.6%)/Male 16 (44.4%)	LipiSperse PEA (150 mg/bid or 300 mg/bid, capsule, oral)	8 weeks, monotherapy
			PEA(600 mg): 58.0 ± 11.0	PEA(600 mg): Female 17 (48.6%)/Male 18 (51.4%)		
			PLB: 55.0 ± 11.0	PLB: Female 22 (55.0%)/Male 18 (45.0%)		
Strobbe et al., 2013 (Italy)	Double-blind	Ocular hypertension (Clinical assessment, IOP)	PEA: 56.8 ± 8.1	PEA: Female 16 (40.0%)/Male 24 (60.0%)	Undeclared PEA formulation (300 mg/bid, tablet, oral)	12 weeks, monotherapy
			PLB: 56.2 ± 10.4	PLB: Female 22 (55.0%)/Male 18 (45.0%)		
Tartaglia et al., 2015 (Italy)	X	Primary dysmenorrhea (Clinical assessment)	PEA: 19,88 ± 2.27	N/A	PEA/transpolydatin (PEA + transpolydatin once/day, tablet, oral; dosage not given)	10 days, monotherapy (from the 24th day of cycle)
			PLB: 19,86 ± 2.35			
Versace et al., 2023 (Italy)	Double-blind	Post-COVID-19 cognitive dyfunction and fatigue (Clinical assessment, PCR nasopharyngeal swab)	PEA: 53.5 ± 10.4	PEA: Female 11 (64.7%)/Male 6 (35.3%)	Co-ultraPEALut (umPEA 700 mg + luteolin 70 mg/bid, microgranules, sublingual)	8 weeks, monotherapy
			PLB: 48.1 ± 10.7	PLB: Female 11 (64.7%)/Male 6 (35.3%)		
Yuan et al., 2014 (China)	Double-blind	Asteatotic eczema (Clinical assessment)	PEA: 51.90 ± 10.9	PEA: Female 30 (100.0%)/Male 0 (0.0%)	PEA/AEA (PEA 0.3% + AEA 0.21% 2 mg/cm^2^/bid, emollient cream, skin application)	4 weeks, monotherapy
			CTRL: 53.43 ± 9.8	CTRL: Female 30 (100.0%)/Male 0 (0.0%)		

AAOS, American Academy of Orthopaedic Surgeons; ACR, American College of Rheumatology; AEA, Anandamide; ALA, Alpha-lipoic acid; ALC, Acetyl-L-Carnitine; BCVA, Best Corrected Visual Acuity; CAM, Confusion Assessment Method; Co-ultraPEALut, Co-ultramicronized PEA plus Luteolin; COD, Chronic olfactory dysfunction; COVID-19, SARS-CoV-2 infection; CPSI, Chronic Prostatitis Symptom Index; ctp, Citalopram; CTRL, Control; DAS, Disease Activity Score; dlx, Duloxetine; DN4, Neuropathic pain diagnostic questionnaire; DOM, Delirium-O-Meter; DSM, Diagnostic and Statistical Manual of Mental Disorders; ENG, Electroneurography; ESHRE, European Society of Human Reproduction and Embryology; GCS, Glasgow Coma Scale; h, hours; GHT, Glaucoma Hemifield Test; ICHD3, International Classification of Headache Disorders, 3rd Edition; IHCC, International Headache Classification Criteria; IOP, Intra-ocular pressure; lth, Lithium; MINI, The Mini International Neuropsychiatric Interview; mPEA, Micronized-PEA; MRI, Magnetic Resonance Imaging; N/A, Not applicable; NIH, National Institutes of Health; NIHSS, National Institutes of Health Stroke Scale; OT, Olfactory training; PCR, Polymerase Chain Reaction; PEA, Palmitoylethanolamide; pgb, Pregabalin; PLB, Placebo; PSA, Prostate-specific antigen; PSQI, Pittsburgh Sleep Quality Index; RDC/TMD, Reasearch Diagnostic Criteria for TMD; rlz, Riluzole; rsp, Risperidone; rTNSS, Reflective Total Nasal Symptom Score; S-LANSS, Self-reported Leeds Assessment of Neuropathic Symptoms and Signs; SCI, Spinal Cord Injury; SCID, Structured Clinical Interview of DSM; SRP, Scaling and root planning; TAU, Treatment as usual; TENS, Transcutaneous electrical nerve stimulation; TMD, Temporo-mandibular Disorder; umPEA, Ultramicronized-PEA.

All included studies underwent critical appraisal. Most studies were classified as having good quality, while two studies were categorized as having fair quality (Costagliola et al., 2014; Albanese et al., 2022), as assessed using the JBI critical appraisal tool for RCTs (Barker et al., 2023). The detailed risk of bias and quality assessment with the corresponding questions for each item and list of RCTs is shown in Table 3.

**Table 3:** Risk of bias and quality assessment (Johanna Briggs Institute tool for Randomized Controlled Trials).

Author, year/Question	Q1	Q2	Q3	Q4	Q5	Q6	Q7	Q8	Q9	Q10	Q11	Q12	Q13	Quality Appraisal
Abedini et al., 2022	Y	Y	Y	Y	Y	Y	Y	Y	Y	Y	Y	Y	Y	Good
Albanese et al., 2022	U	U	Y	U	U	Y	U	Y	Y	Y	Y	Y	N	Fair
Andresen et al., 2016	Y	Y	Y	Y	Y	Y	U	Y	Y	Y	Y	Y	Y	Good
Bacci et al., 2011	Y	U	Y	Y	N	Y	U	Y	Y	U	Y	Y	Y	Good
Bonzanino et al., 2024	Y	N	Y	N	N	Y	N	Y	Y	U	Y	Y	Y	Good
Briskey et al., 2023	Y	Y	Y	Y	Y	Y	Y	Y	Y	U	Y	Y	Y	Good
Briskey et al., 2024	Y	Y	Y	Y	U	Y	Y	Y	Y	U	Y	Y	Y	Good
Campolo et al., 2021	Y	U	Y	U	U	Y	Y	Y	Y	Y	Y	Y	Y	Good
Cantone et al., 2024	Y	Y	Y	U	Y	Y	Y	Y	Y	U	Y	Y	Y	Good
Cobellis et al., 2011	Y	Y	Y	Y	Y	Y	U	Y	Y	Y	Y	Y	Y	Good
Coraci et al., 2018	Y	Y	Y	Y	U	Y	U	Y	Y	U	Y	Y	U	Good
Costagliola et al., 2014	U	N	U	N	N	Y	N	Y	Y	Y	Y	Y	Y	Fair
Cremon et al., 2017	Y	Y	Y	Y	Y	Y	Y	Y	Y	Y	Y	Y	Y	Good
D'Ascanio et al., 2021	Y	N	N	Y	N	Y	N	Y	Y	Y	Y	Y	Y	Good
Di Nardo et al., 2024	Y	Y	Y	Y	Y	Y	Y	Y	Y	Y	Y	Y	Y	Good
Di Stadio et al., 2022	Y	Y	Y	Y	Y	Y	Y	Y	Y	Y	Y	Y	Y	Good
Di Stadio et al., 2023a	U	Y	N	Y	Y	Y	Y	Y	Y	Y	Y	Y	Y	Good
Di Stadio et al., 2023b	Y	Y	Y	Y	Y	N	Y	Y	Y	Y	Y	Y	Y	Good
Evangelista et al., 2018	Y	N	Y	N	N	Y	N	Y	Y	Y	Y	Y	Y	Good
Faig-Marti and Martinez-Catassus, 2017 Faig-Marti and Martinez-Catassus, 2020	Y	Y	Y	Y	Y	Y	U	Y	Y	Y	Y	Y	Y	Good
Gagliano et al., 2011	Y	Y	Y	Y	Y	Y	Y	Y	Y	Y	Y	Y	Y	Good
Germini et al., 2017	Y	N/A	N/A	Y	Y	N/A	Y	Y	Y	N/A	N/A	Y	Y	Good
Ghazizadeh-Hashemi et al., 2018	Y	Y	Y	Y	Y	Y	Y	Y	Y	Y	Y	Y	Y	Good
Giammusso et al., 2017	Y	Y	Y	Y	Y	N	U	Y	Y	Y	Y	Y	Y	Good
Guida et al., 2010	Y	Y	Y	Y	Y	N	U	Y	Y	Y	Y	Y	Y	Good
Isola et al., 2021	Y	N	Y	N	N	Y	Y	Y	Y	Y	Y	Y	Y	Good
Kadanangode et al., 2023	Y	N	Y	N	N	Y	N	Y	Y	N	Y	Y	Y	Good
Khalaj et al., 2018	Y	U	Y	Y	Y	Y	U	Y	Y	Y	Y	Y	Y	Good
Lunardelli et al., 2019	Y	Y	Y	Y	N	Y	N	Y	Y	Y	Y	Y	Y	Good
Marini et al., 2012	Y	Y	Y	Y	Y	N	Y	Y	Y	Y	Y	Y	Y	Good
Masek et al., 1974	Y	Y	U	Y	Y	Y	U	Y	N	Y	Y	Y	Y	Good
Murina et al., 2013	Y	Y	Y	Y	Y	Y	Y	Y	Y	Y	Y	Y	Y	Good
Orefice et al., 2016	Y	Y	Y	Y	Y	Y	U	Y	Y	Y	Y	Y	Y	Good
Ottaviani et al., 2019	Y	Y	N	Y	Y	Y	Y	Y	Y	Y	Y	Y	Y	Good
Palma et al., 2016	Y	Y	Y	Y	U	Y	U	Y	Y	U	Y	Y	Y	Good
Pickering et al., 2022	Y	Y	Y	Y	Y	Y	Y	Y	Y	Y	Y	Y	Y	Good
Rao et al., 2021	Y	Y	Y	Y	Y	Y	U	Y	Y	Y	Y	Y	Y	Good
Rao et al., 2023a	Y	Y	Y	Y	Y	Y	Y	Y	Y	N	Y	Y	Y	Good
Rao et al., 2023b	Y	Y	Y	U	U	Y	U	Y	Y	N	Y	Y	Y	Good
Rossi et al., 2020	Y	Y	Y	N	N	N	Y	Y	Y	Y	Y	Y	Y	Good
Salaffi et al., 2023	Y	Y	Y	N	N	Y	N	Y	Y	N	Y	Y	Y	Good
Salehi et al., 2022	Y	Y	Y	Y	Y	Y	U	Y	Y	Y	Y	Y	Y	Good
Steels et al., 2019	Y	Y	Y	Y	Y	Y	U	Y	Y	Y	Y	Y	Y	Good
Strobbe et al., 2013	Y	Y	Y	Y	Y	Y	Y	Y	Y	Y	Y	Y	Y	Good
Tartaglia et al., 2015	Y	Y	Y	U	U	U	U	Y	Y	Y	Y	Y	Y	Good
Versace et al., 2023	N	Y	Y	Y	Y	Y	U	Y	Y	Y	Y	Y	Y	Good
Yuan et al., 2014	Y	U	Y	Y	Y	Y	U	Y	Y	Y	Y	Y	Y	Good

Qn, Question; Y, Yes; N, No; U, Unclear; N/A, Not applicable.

### 3.2 Effect of Palmitoylethanolamide supplementation on neuropsychiatric disturbances across human conditions

Fifteen RCTs addressed this area (Table 1) using similar but not overlapping methodologies in terms of clinical condition of interest [bipolar affective disorder (BPAD) (Abedini et al., 2022), spinal cord injury (SCI) (Andresen et al., 2016), traumatic brain injury (TBI) (Campolo et al., 2021), carpal tunnel syndrome (CTS) (Evangelista et al., 2018), primary open angle glaucoma/ocular hypertension (POAG/OH) (Gagliano et al., 2011), major depressive disorder (MDD) (Ghazizadeh-Hashemi et al., 2018), autism spectrum disorder (ASD) (Khalaj et al., 2018), post-operative delirium (POD) (Lunardelli et al., 2019), relapsing-remitting multiple sclerosis (RR-MS) (Orefice et al., 2016), diabetes (Pickering et al., 2022), disturbed sleep pattern (DSP) (Rao et al., 2021), schizophrenia (SCZ) (Salehi et al., 2022), knee osteoarthritis (OA) (Steels et al., 2019), SARS-Cov-2 (COVID-19) infection (Versace et al., 2023), acute ischemic stroke (AIS) (Bonzanino et al., 2024)], PEA formulation [undeclared (Abedini et al., 2022; Gagliano et al., 2011; Khalaj et al., 2018; Salehi et al., 2022), ultramicronized-PEA (umPEA) (Andresen et al., 2016; Evangelista et al., 2018; Ghazizadeh-Hashemi et al., 2018; Orefice et al., 2016), Co-ultramicronized PEA plus Luteolin (Co-ultraPEALut) (Campolo et al., 2021; Lunardelli et al., 2019; Versace et al., 2023; Bonzanino et al., 2024), LipiSperse PEA (Pickering et al., 2022; Rao et al., 2021; Steels et al., 2019)], PEA intake route (oral capsule (Abedini et al., 2022; Ghazizadeh-Hashemi et al., 2018; Khalaj et al., 2018; Pickering et al., 2022; Rao et al., 2021; Salehi et al., 2022; Steels et al., 2019), sublingual microgranules (Andresen et al., 2016; Evangelista et al., 2018; Versace et al., 2023), oral tablet (Campolo et al., 2021; Evangelista et al., 2018; Gagliano et al., 2011; Lunardelli et al., 2019; Orefice et al., 2016), oral suspension (Bonzanino et al., 2024)), PEA dosage (600 mg/bid (Abedini et al., 2022; Andresen et al., 2016; Evangelista et al., 2018; Ghazizadeh-Hashemi et al., 2018; Khalaj et al., 2018), 700 mg/bid (Campolo et al., 2021; Lunardelli et al., 2019; Salehi et al., 2022; Versace et al., 2023; Bonzanino et al., 2024), 600 mg/day (Evangelista et al., 2018; Gagliano et al., 2011; Orefice et al., 2016), 300 mg/bid (Pickering et al., 2022; Steels et al., 2019), 150 mg/bid (Rao et al., 2021; Steels et al., 2019)), PEA period of exposure (6 weeks (Abedini et al., 2022; Ghazizadeh-Hashemi et al., 2018), 12 weeks (Andresen et al., 2016; Bonzanino et al., 2024), 26 weeks (Campolo et al., 2021), 20 weeks (Evangelista et al., 2018), 8 weeks (Gagliano et al., 2011; Pickering et al., 2022; Rao et al., 2021; Salehi et al., 2022; Steels et al., 2019; Versace et al., 2023), 10 weeks (Khalaj et al., 2018), 3 days (Lunardelli et al., 2019), 53 weeks (Orefice et al., 2016)), PEA use as monotherapy (Evangelista et al., 2018; Gagliano et al., 2011; Lunardelli et al., 2019; Orefice et al., 2016; Pickering et al., 2022; Rao et al., 2021; Steels et al., 2019; Versace et al., 2023) or add-on therapy (Abedini et al., 2022; Andresen et al., 2016; Campolo et al., 2021; Ghazizadeh-Hashemi et al., 2018; Khalaj et al., 2018; Salehi et al., 2022; Bonzanino et al., 2024), level of concealment (double-blind (Abedini et al., 2022; Andresen et al., 2016; Gagliano et al., 2011; Ghazizadeh-Hashemi et al., 2018; Khalaj et al., 2018; Orefice et al., 2016; Rao et al., 2021; Salehi et al., 2022; Steels et al., 2019; Versace et al., 2023), single-blind (Campolo et al., 2021; Lunardelli et al., 2019), open-label (Evangelista et al., 2018; Bonzanino et al., 2024), quadruple-blind (Pickering et al., 2022)), and comparison to placebo (Abedini et al., 2022; Andresen et al., 2016; Gagliano et al., 2011; Ghazizadeh-Hashemi et al., 2018; Khalaj et al., 2018; Orefice et al., 2016; Pickering et al., 2022; Rao et al., 2021; Salehi et al., 2022; Steels et al., 2019; Versace et al., 2023) or control treatments (Campolo et al., 2021; Evangelista et al., 2018; Lunardelli et al., 2019; Bonzanino et al., 2024). Notably, PEA was well-tolerated throughout the studies, showing no signs of extrapyramidal symptoms (Abedini et al., 2022; Salehi et al., 2022) or any other relevant adverse events (AEs) (Abedini et al., 2022; Andresen et al., 2016; Evangelista et al., 2018; Gagliano et al., 2011; Ghazizadeh-Hashemi et al., 2018; Khalaj et al., 2018; Lunardelli et al., 2019; Pickering et al., 2022; Rao et al., 2021; Salehi et al., 2022; Steels et al., 2019; Bonzanino et al., 2024) during the entire period of administration.

#### 3.2.1 Effect of Palmitoylethanolamide supplementation on mood, anxiety, and stress

Overall, studies exploring PEA ability to improve mood disturbances compared to placebo or control treatment suggested its efficacy as add-on therapy in reducing either depressive symptoms in citalopram-treated MDD patients (Ghazizadeh-Hashemi et al., 2018) and acute manic symptoms in lithium- and risperidone-treated BPAD patients (Abedini et al., 2022), as well as monotherapy in reducing depressive symptoms in diabetic patients with peripheral neuropathy (Pickering et al., 2022). Contrastingly, no significant reduction of depressive symptoms emerged in PEA-treated BPAD (Abedini et al., 2022), SCI (Andresen et al., 2016), TBI (Campolo et al., 2021), SCZ (Salehi et al., 2022), and knee OA (Steels et al., 2019) patients, in comparison to placebo or control treatment. A limited number of studies examined PEA effects on anxiety (Andresen et al., 2016; Pickering et al., 2022; Steels et al., 2019) and stress (Pickering et al., 2022; Steels et al., 2019), with benefits observed specifically on anxiety symptoms among knee OA patients (Steels et al., 2019) and a trend towards reduced stress levels among PEA-treated diabetic patients (Pickering et al., 2022). Lastly, no increase in the occurrence of mood disorders and anxiety, monitored as adverse events, was observed among PEA-treated patients with POAG/OH (Gagliano et al., 2011).

#### 3.2.2 Effect of Palmitoylethanolamide supplementation on attention, awareness, and cognition

Five RCTs explored the selective effects of PEA across conditions affecting higher-level cognitive functions (Campolo et al., 2021; Lunardelli et al., 2019; Orefice et al., 2016; Versace et al., 2023; Bonzanino et al., 2024). A single trial demonstrated the superiority of PEA compared to placebo add-on to treatment as usual (TAU; i.e., antiplatelet agents, anticoagulants, antiepileptic medications) in the recovery of TBI-related deficits in working memory and executive functions (Campolo et al., 2021). PEA add-on to TAU resulted in an increased number of thrombolysis-treated AIS patients able to undergo cognitive evaluations after the stroke event, as well as an improvement in cognitive performances, although the superiority over the comparator was not proven (Bonzanino et al., 2024). Also, PEA monotherapy proved to be effective in reducing both the incidence and maximum severity of POD, although not affecting POD duration, in comparison to control treatment (Lunardelli et al., 2019). On the contrary, no difference between PEA and placebo was observed over measures of calculation ability and auditory information processing speed and flexibility among RR-MS patients, despite reports of subjectively improved cognitive function and health (Orefice et al., 2016). Finally, PEA monotherapy enhanced measures of Gamma-aminobutyric acid (GABA)_B_ergic activity and synaptic plasticity among patients experiencing long-term post-COVID-19 fatigue and cognitive sequelae (e.g., forgetfulness, cloudiness, difficulty in focusing), although it did not significantly impact clinical measures of cognitive performance (Versace et al., 2023).

#### 3.2.3 Effect of Palmitoylethanolamide supplementation on sleep

Accumulating studies have been parsing PEA effects on several sleep domains across different clinical conditions, including SCI (Andresen et al., 2016), CTS (Evangelista et al., 2018), diabetes (Pickering et al., 2022), and knee OA (Steels et al., 2019), converging towards the evidence of improved sleep-wake rhythm or sleep adequacy (Evangelista et al., 2018; Pickering et al., 2022), sleep latency (Evangelista et al., 2018), sleep-related troubles in daily activities (e.g., shortness of breath or headache) or daytime somnolence (Evangelista et al., 2018; Pickering et al., 2022), and sleep duration (Evangelista et al., 2018). In contrast, mixed or not significant findings emerged regarding PEA efficacy on measures of sleep quality (Evangelista et al., 2018; Steels et al., 2019), sleep quantity (Pickering et al., 2022), and sleep disturbances, snoring, or insomnia (Andresen et al., 2016; Evangelista et al., 2018; Pickering et al., 2022). Additionally, PEA was exclusively effective on self-monitored sleep onset latency among subjects with primary DSP, while it did not have a significant impact on other sleep measures (Rao et al., 2021). Notably, there were no observed sleep disturbances reported as adverse events in PEA-treated patients with POAG/OH (Gagliano et al., 2011).

#### 3.2.4 Effect of Palmitoylethanolamide supplementation on psychotic symptoms and autistic behaviors

A single study demonstrated the beneficial effects of PEA adjunctive therapy on residual negative psychotic symptoms and general psychopathology measures in risperidone-treated SCZ patients, although not resulting in improved positive psychotic symptoms in comparison to placebo (Salehi et al., 2022). Results from an RCT among risperidone-treated autistic children yielded the superiority of PEA add-on compared to placebo in the treatment of core symptoms of ASD, particularly in the domains of irritability, hyperactivity, and inappropriate speech, but not stereotypic behavior, either in terms of symptom improvement or response rate (Khalaj et al., 2018).

### 3.3 Effect of Palmitoylethanolamide supplementation on sensory and/or motor neurological disturbances across human conditions

Altogether, seventeen RCTs explored this topic, using similar but not overlapping methodologies in terms of condition of interest [SCI (Andresen et al., 2016), migraine (Briskey et al., 2024), TBI (Campolo et al., 2021), CTS (Evangelista et al., 2018; Coraci et al., 2018; Faig-Marti and Martinez-Catassus, 2017, 2020), post-COVID-19 olfactory impairment (D'Ascanio et al., 2021; Di Stadio et al., 2022; Di Stadio et al., 2023a; Cantone et al., 2024; Di Stadio et al., 2023b), compressive-type lumbar sciatica (Guida et al., 2010), RR-MS (Orefice et al., 2016), burning mouth syndrome (BMS) (Ottaviani et al., 2019), amyotrophic lateral sclerosis (ALS) (Palma et al., 2016), diabetes (Pickering et al., 2022), AIS (Bonzanino et al., 2024)], PEA formulation [umPEA (Andresen et al., 2016; Evangelista et al., 2018; Orefice et al., 2016; Ottaviani et al., 2019; Palma et al., 2016), Co-ultraPEALut (Campolo et al., 2021; Bonzanino et al., 2024; D'Ascanio et al., 2021; Di Stadio et al., 2022; Di Stadio et al., 2023a; Cantone et al., 2024; Di Stadio et al., 2023b), undeclared (Coraci et al., 2018; Faig-Marti and Martinez-Catassus, 2017, 2020), micronized-PEA (mPEA) (Guida et al., 2010), LipiSperse PEA (Pickering et al., 2022; Briskey et al., 2024), Co-ultraPEALut/Alpha-lipoic acid (ALA) (Cantone et al., 2024)], PEA intake route (sublingual microgranules (Andresen et al., 2016; Evangelista et al., 2018; Ottaviani et al., 2019), oral tablet (Campolo et al., 2021; Evangelista et al., 2018; Orefice et al., 2016; Coraci et al., 2018; D'Ascanio et al., 2021; Di Stadio et al., 2022; Di Stadio et al., 2023a; Faig-Marti and Martinez-Catassus, 2017; Faig-Marti and Martinez-Catassus, 2020; Palma et al., 2016), oral capsule (Pickering et al., 2022; Guida et al., 2010; Briskey et al., 2024), oral sachet (Cantone et al., 2024; Di Stadio et al., 2023b), oral suspension (Bonzanino et al., 2024)), PEA dosage (600 mg/bid (Andresen et al., 2016; Evangelista et al., 2018; Ottaviani et al., 2019; Palma et al., 2016), 700 mg/bid (Campolo et al., 2021; Bonzanino et al., 2024; D'Ascanio et al., 2021; Di Stadio et al., 2022; Di Stadio et al., 2023a), 600 mg/day (Evangelista et al., 2018; Orefice et al., 2016; Coraci et al., 2018; Faig-Marti and Martinez-Catassus, 2017, 2020), 300 mg/day (Guida et al., 2010), 300 mg/bid (Pickering et al., 2022; Guida et al., 2010; Briskey et al., 2024), 700 mg/day (Cantone et al., 2024; Di Stadio et al., 2023b)), PEA period of exposure (12 weeks (Andresen et al., 2016; Bonzanino et al., 2024; Di Stadio et al., 2022; Di Stadio et al., 2023a; Cantone et al., 2024; Di Stadio et al., 2023b), 8 h (Briskey et al., 2024), 26 weeks (Campolo et al., 2021), 4 weeks (Coraci et al., 2018; D'Ascanio et al., 2021), 20 weeks (Evangelista et al., 2018), 8 weeks (Pickering et al., 2022; Faig-Marti and Martinez-Catassus, 2017, 2020; Ottaviani et al., 2019), 3 weeks (Guida et al., 2010), 53 weeks (Orefice et al., 2016), 24 weeks (Palma et al., 2016)), PEA use as monotherapy (Evangelista et al., 2018; Orefice et al., 2016; Pickering et al., 2022; Coraci et al., 2018; D'Ascanio et al., 2021; Di Stadio et al., 2022; Di Stadio et al., 2023a; Faig-Marti and Martinez-Catassus, 2017; Faig-Marti and Martinez-Catassus, 2020; Guida et al., 2010; Ottaviani et al., 2019; Cantone et al., 2024; Di Stadio et al., 2023b; Briskey et al., 2024) or add-on therapy (Andresen et al., 2016; Campolo et al., 2021; Bonzanino et al., 2024; Palma et al., 2016; Di Stadio et al., 2023b), level of concealment (double-blind (Andresen et al., 2016; Orefice et al., 2016; Coraci et al., 2018; Di Stadio et al., 2022; Di Stadio et al., 2023a; Faig-Marti and Martinez-Catassus, 2017; Faig-Marti and Martinez-Catassus, 2020; Guida et al., 2010; Ottaviani et al., 2019; Briskey et al., 2024), single-blind (Campolo et al., 2021; D'Ascanio et al., 2021; Palma et al., 2016; Cantone et al., 2024; Di Stadio et al., 2023b), open-label (Evangelista et al., 2018; Bonzanino et al., 2024), quadruple-blind (Pickering et al., 2022)), and comparison to placebo (Andresen et al., 2016; Orefice et al., 2016; Pickering et al., 2022; Coraci et al., 2018; Di Stadio et al., 2022; Di Stadio et al., 2023a; Faig-Marti and Martinez-Catassus, 2017; Faig-Marti and Martinez-Catassus, 2020; Guida et al., 2010; Ottaviani et al., 2019; Cantone et al., 2024; Di Stadio et al., 2023b; Briskey et al., 2024) or control treatments (Campolo et al., 2021; Evangelista et al., 2018; Bonzanino et al., 2024; D'Ascanio et al., 2021; Palma et al., 2016; Cantone et al., 2024). PEA was well-tolerated throughout the studies, showing no relevant AEs during the entire period of administration (Andresen et al., 2016; Evangelista et al., 2018; Pickering et al., 2022; Bonzanino et al., 2024; Di Stadio et al., 2022; Faig-Marti and Martinez-Catassus, 2017; Faig-Marti and Martinez-Catassus, 2020; Guida et al., 2010; Ottaviani et al., 2019; Briskey et al., 2024).

#### 3.3.1 Effect of Palmitoylethanolamide supplementation on neuropathic pain

The major strand of RCTs reported on PEA effects over neuropathic pain across several conditions, including CTS (Evangelista et al., 2018; Coraci et al., 2018; Faig-Marti and Martinez-Catassus, 2017, 2020), SCI (Andresen et al., 2016), lumbar sciatica (Guida et al., 2010), BMS (Ottaviani et al., 2019), and diabetes (Pickering et al., 2022). Among the three RCTs investigating PEA effects on neuropathic pain in CTS patients, PEA was found to significantly improve ultrasound (Coraci et al., 2018) and subjective measures of symptom severity and functional status (Faig-Marti and Martinez-Catassus, 2017, 2020; Faig-Marti and Martinez-Catassus, 2017, 2020) overtime, although yielding conflicting results in terms of hyperalgesia control (Evangelista et al., 2018; Faig-Marti and Martinez-Catassus, 2017, 2020), time without paresthesia during wrist flexion (Evangelista et al., 2018; Faig-Marti and Martinez-Catassus, 2017, 2020), and electrophysiological assessments (Coraci et al., 2018; Faig-Marti and Martinez-Catassus, 2017, 2020). Patients with lumbar sciatica treated with PEA experienced significantly greater reduction in pain intensity and improvement in disability measures compared to placebo, with effects increasing in a dose-dependent manner (Guida et al., 2010). Neuropathic pain, perceived as burning sensation, was also improved among PEA-treated BMS patients compared to those treated with placebo, and pain reduction persisted even 4 months after PEA discontinuation (Ottaviani et al., 2019). An RCT investigated the efficacy of PEA in managing pain associated with diabetic peripheral neuropathy (PNP), revealing a significant decrease in overall pain severity, pain interference with life activities, superficial and deep pain, paroxysmal and evoked pain, and paresthesia (Pickering et al., 2022). Finally, a study examining the effects of PEA on symptoms of traumatic or non-traumatic SCI found a significantly larger decrease in the use of rescue medications compared to placebo, although there was no impact on sensory examination, global impression, neuropathic pain relief, pain-associated unpleasantness, pain interference with life activities, and health-related quality of life (Andresen et al., 2016).

#### 3.3.2 Effect of Palmitoylethanolamide supplementation on pain associated with migraine

A single RCT addressed PEA effects in migraine patients instructed to take either PEA or placebo at pain onset and 2 h later in case of persisting episode. The study found PEA superiority to placebo, with more resolved headaches after 2 h, more resolved headaches after 8 h, more resolved migraines of moderate severity at onset, lower pain scores at 1.5 h, lower pain scores at 4 h, and reduced need for rescue medications (Briskey et al., 2024).

#### 3.3.3 Effect of Palmitoylethanolamide supplementation on altered olfactory function

Five studies investigated the effects of PEA monotherapy on olfactory impairment recovery in post-COVID-19 patients, both prior to (Di Stadio et al., 2022, 2023a), after (D'Ascanio et al., 2021; Cantone et al., 2024), or alongside olfactory training (Di Stadio et al., 2023b), using overlapping methodologies in terms of PEA formulation (i.e., Co-ultraPEALut, Co-ultraPEALut/ALA) and sensory function assessment. Consistent improvements emerged in global anosmia/hyposmia scores among patients treated with PEA, also showing its superiority against comparators (D'Ascanio et al., 2021; Di Stadio et al., 2022; Di Stadio et al., 2023a; Cantone et al., 2024; Di Stadio et al., 2023b), as well as in terms of variation in anosmia/hyposmia scores (Di Stadio et al., 2022), and proportion of patients recovering from anosmia (Di Stadio et al., 2022, 2023a). A study out of two (Cantone et al., 2024) showed significantly higher parosmia resolution rates among Co-ultraPEALut- and Co-ultraPEALut/ALA-treated patients compared to those treated with ALA monotherapy, and among Co-ultraPEALut/ALA-treated patients compared to those treated with Co-ultraPEALut or placebo (Di Stadio et al., 2023a; Cantone et al., 2024).

#### 3.3.4 Effect of Palmitoylethanolamide supplementation on mobility, spasticity, and sensory-motor deficits

Studies exploring this area produced mixed findings regarding the impact of PEA on motor neurological symptoms. PEA add-on effect did not significantly differ from placebo in terms of stiffness, spasms, and other descriptors of spasticity among SCI patients (Andresen et al., 2016), nor modulated motor disability progression in β1a-IFN-treated patients with RR-MS (Orefice et al., 2016). On the other hand, self-reported spasticity was higher in PEA-treated SCI patients compared to those on placebo (Andresen et al., 2016). Also, patients with moderate TBI who were treated with PEA add-on to TAU showed significant ameliorations in their independence and mobility in daily activities, relative to their baseline condition, although superiority over control therapy remains to be clarified (Campolo et al., 2021). A single study conducted among thrombolysis-treated AIS patients showed that PEA initiation as add-on to TAU (i.e., alteplase) within the first 72 h after the ischemic event led to improved neurological deficits overtime (Bonzanino et al., 2024). Additionally, PEA-treated patients experienced greater improvements in mobility during daily activities and functional independence, showing significantly better outcomes than the comparator group by the end of the treatment (Bonzanino et al., 2024).

#### 3.3.5 Effect of Palmitoylethanolamide supplementation on altered muscle force and respiratory capacity

Results from a single study interrogating the effect of PEA add-on to riluzole compared to riluzole alone in ALS patients converged towards higher proportion of survivors among PEA-treated patients. Indeed, a slower decline of pulmonary function over time emerged, assessed as percentage of forced vital capacity (FVC%) and bulbar and respiratory functions measurements (Palma et al., 2016).

### 3.4 Effect of Palmitoylethanolamide supplementation on somatic disturbances across human conditions

Thirteen studies appraised this area (Orefice et al., 2016; Steels et al., 2019; Bacci et al., 2011; Germini et al., 2017; Isola et al., 2021; Marini et al., 2012; Masek et al., 1974; Yuan et al., 2014; Briskey et al., 2023; Kadanangode et al., 2023; Rao et al., 2023a, 2023b; Salaffi et al., 2023), using similar but not overlapping methodologies in terms of condition of interest [impacted lower third molar (ILTM) (Bacci et al., 2011), allergic rhinitis (AR) (Briskey et al., 2023), chronic back/joints/limbs pain (Germini et al., 2017), periodontitis (Isola et al., 2021), arthritis (Kadanangode et al., 2023), temporomandibular joint OA (Marini et al., 2012), upper respiratory tract infections (URTI) (Masek et al., 1974; Rao et al., 2023a), RR-MS (Orefice et al., 2016), atopic eczema (Rao et al., 2023b), fibromyalgia (FM) (Salaffi et al., 2023), knee OA (Steels et al., 2019), asteatotic eczema (Yuan et al., 2014)], PEA formulation (mPEA (Bacci et al., 2011), LipiSperse PEA (Steels et al., 2019; Briskey et al., 2023; Rao et al., 2023a, 2023b), umPEA (Orefice et al., 2016; Germini et al., 2017), PEA/baicalin (Isola et al., 2021), PEA/astaxanthin (Kadanangode et al., 2023), undeclared (Marini et al., 2012), Impulsin (Masek et al., 1974), PEA/Acetyl-L-Carnitine (ALC) (Salaffi et al., 2023), PEA/AEA (Yuan et al., 2014)), PEA intake route (oral tablet (Orefice et al., 2016; Bacci et al., 2011; Germini et al., 2017; Marini et al., 2012; Masek et al., 1974; Kadanangode et al., 2023; Salaffi et al., 2023), oral capsule (Steels et al., 2019; Isola et al., 2021; Briskey et al., 2023; Rao et al., 2023a), skin-applied moisturizing cream (Rao et al., 2023b), skin-applied emollient cream (Yuan et al., 2014)), PEA dosage (300 mg/bid (Steels et al., 2019; Bacci et al., 2011; Marini et al., 2012; Rao et al., 2023a), 300 mg/day (Briskey et al., 2023), 600 mg/bid (Germini et al., 2017; Salaffi et al., 2023), 800 mg/bid (Isola et al., 2021; Kadanangode et al., 2023), 300 mg + 600 mg/day (Marini et al., 2012), 600 mg/tid (Masek et al., 1974), 600 mg/day (Orefice et al., 2016), 150 mg/bid (Steels et al., 2019), 1.5% concentrated cream/bid (Rao et al., 2023b), 0.3% 2 mg/cm^2^/bid (Yuan et al., 2014)), PEA period of exposure (2 weeks (Bacci et al., 2011; Marini et al., 2012; Briskey et al., 2023), 3 weeks (Germini et al., 2017), 5 days (Isola et al., 2021), 12 weeks (Kadanangode et al., 2023; Rao et al., 2023a; Salaffi et al., 2023), 12 days (Masek et al., 1974), 53 weeks (Orefice et al., 2016), 8 weeks (Steels et al., 2019), 4 weeks (Yuan et al., 2014; Rao et al., 2023b)), PEA use as monotherapy (Orefice et al., 2016; Steels et al., 2019; Bacci et al., 2011; Germini et al., 2017; Marini et al., 2012; Masek et al., 1974; Yuan et al., 2014; Briskey et al., 2023; Rao et al., 2023a, 2023b) or add-on therapy (Isola et al., 2021; Kadanangode et al., 2023; Salaffi et al., 2023), level of concealment (single-blind (Bacci et al., 2011), double-blind (Orefice et al., 2016; Steels et al., 2019; Germini et al., 2017; Isola et al., 2021; Masek et al., 1974; Yuan et al., 2014; Briskey et al., 2023; Rao et al., 2023a, 2023b), open-label (Kadanangode et al., 2023; Salaffi et al., 2023), triple-blind (Marini et al., 2012)), and comparison to placebo (Orefice et al., 2016; Steels et al., 2019; Germini et al., 2017; Masek et al., 1974; Briskey et al., 2023; Rao et al., 2023a) or control treatments (Bacci et al., 2011; Isola et al., 2021; Marini et al., 2012; Yuan et al., 2014; Kadanangode et al., 2023; Rao et al., 2023b; Salaffi et al., 2023). A minority of studies systematically reported on PEA good tolerability during the entire observation period (Steels et al., 2019; Bacci et al., 2011; Germini et al., 2017; Isola et al., 2021; Marini et al., 2012; Yuan et al., 2014; Kadanangode et al., 2023; Rao et al., 2023a, 2023b; Salaffi et al., 2023), only mentioning isolated cases of drowsiness (Bacci et al., 2011), palpitations (Bacci et al., 2011), diarrhea (Germini et al., 2017; Rao et al., 2023a), skin rash (Rao et al., 2023a), dizziness (Salaffi et al., 2023), nausea (Salaffi et al., 2023), blurred vision (Salaffi et al., 2023), sleepiness (Salaffi et al., 2023), and dry mouth (Salaffi et al., 2023).

#### 3.4.1 Effect of Palmitoylethanolamide supplementation on joints, back, limbs, and chronic widespread pain and altered function

Five RCTs explored the efficacy of PEA in this group of disturbances. Three studies addressing PEA effect on temporomandibular (Marini et al., 2012), knee (Steels et al., 2019; Kadanangode et al., 2023), and hip (Kadanangode et al., 2023) OA (Steels et al., 2019; Marini et al., 2012) or isolated arthritis (Kadanangode et al., 2023) pointed towards the evidence of a greater reduction of total and average pain measures (Steels et al., 2019; Marini et al., 2012; Kadanangode et al., 2023), decreased worst and least pain levels (Marini et al., 2012), and improved measures of joint and physical stiffness, joint function, and joint mobility range (Steels et al., 2019; Marini et al., 2012; Kadanangode et al., 2023), in comparison to baseline scores (Kadanangode et al., 2023), placebo (Steels et al., 2019), and nonsteroidal anti-inflammatory drugs (NSAIDs) (Marini et al., 2012). A pilot RCT conducted in single elderly outpatients (N-of-1 design) evaluated the effectiveness of PEA monotherapy on chronic non-cancer, non-ischemic pain in the back, joints, or limbs, showing a statistically significant improvement in pain intensity and function impairment in three out of seven patients completing the trial compared to placebo (Germini et al., 2017). Lastly, a single RCT explored the effects of PEA add-on to duloxetine plus pregabalin compared to duloxetine plus pregabalin alone among FM patients, showing favorable effects on widespread nociplastic pain, with improvements in quality of life measures (Salaffi et al., 2023).

#### 3.4.2 Effect of Palmitoylethanolamide supplementation on dental and periodontal inflammation

The efficacy of PEA has been tested in the context of orthodontic treatments, encompassing both surgical (Bacci et al., 2011) and non-surgical (Isola et al., 2021) approaches. In surgical patients who underwent lower third molar extraction, treatment with PEA led to a significant reduction in postoperative pain and delayed edema onset, but not improved swelling severity, compared to control group (Bacci et al., 2011). Similarly, PEA administration following scaling and root planing (SRP) in patients with periodontitis significantly improved both short-term postoperative pain relief and long-term outcomes, including clinical attachment level, probing depth, and bleeding on probing, compared to control group (Isola et al., 2021).

#### 3.4.3 Effect of Palmitoylethanolamide supplementation on respiratory tract infections and allergy symptoms

Two studies investigated PEA effects on patients with URTI, compared to placebo, indicating a significant reduction in episodes of fever (Masek et al., 1974), headache (Masek et al., 1974), sore throat (Masek et al., 1974; Rao et al., 2023a), nasal congestion (Masek et al., 1974), nasal discharge (Masek et al., 1974), cough (Masek et al., 1974; Rao et al., 2023a), and a tendency to improved hoarseness (Rao et al., 2023a) and ability to breathe easily (Rao et al., 2023a), as well as a decrease in the number of total patients (Masek et al., 1974), in-patients (Masek et al., 1974), out-patients (Masek et al., 1974), and patients sick at least once (Rao et al., 2023a). No consistent improvements emerged in terms of illness duration (Masek et al., 1974; Rao et al., 2023a). A single RCT reported on the impact of PEA in AR, indicating beneficial effects on measures of allergy symptoms over time (e.g., nasal congestion, sneezing, itchy nose, runny nose), in both PEA-treated and placebo-treated patients, although with no significant difference between groups (Briskey et al., 2023).

#### 3.4.4 Effect of Palmitoylethanolamide supplementation on cutaneous pain, erythema, and eczema

Three RCTs examined PEA effects on dermatologic issues. PEA significantly improved pain sensation in β1a-IFN cutaneous injection site among RR-MS patients compared to placebo (Orefice et al., 2016). PEA-treated patients with asteatotic eczema experienced improved skin scaling, dryness, itching, and current perception threshold (CPT), compared to a control emollient product, while no effects were observed on skin integrity (Yuan et al., 2014). In both cases, PEA did not impact erythema features (Orefice et al., 2016; Yuan et al., 2014), especially its width (Orefice et al., 2016). A third RCT exploring PEA effects in patients with atopic dermatitis showed significant improvements over time on measures of symptom severity, scratches, and itchiness, also yielding its superiority to a standard moisturizer on measures of redness, dryness, and total symptom scores. No significant impact emerged on quality-of-life measures (Rao et al., 2023b).

### 3.5 Effect of Palmitoylethanolamide supplementation on visceral disturbances across human conditions

Eleven studies addressed this area, using similar but not overlapping methodologies in terms of condition of interest [endometriosis (EMS) (Cobellis et al., 2011), irritable bowel syndrome (IBS) (Cremon et al., 2017; Di Nardo et al., 2024), POAG (Gagliano et al., 2011; Rossi et al., 2020), chronic prostatitis/chronic pelvic pain syndrome (CP/CPPS) (Giammusso et al., 2017), isolated vestibulodynia (Murina et al., 2013), normal tension glaucoma (NTG) (Rossi et al., 2020; Costagliola et al., 2014), isolated OH (Strobbe et al., 2013), primary dysmenorrhea (Tartaglia et al., 2015)], PEA formulation [mPEA/transpolydatin (Cobellis et al., 2011; Murina et al., 2013; Tartaglia et al., 2015), mPEA/polydatin (Cremon et al., 2017; Di Nardo et al., 2024), undeclared (Gagliano et al., 2011; Strobbe et al., 2013), PEA/ALA (Giammusso et al., 2017), umPEA (Rossi et al., 2020; Costagliola et al., 2014)], PEA intake route (oral tablet (Gagliano et al., 2011; Cobellis et al., 2011; Cremon et al., 2017; Murina et al., 2013; Rossi et al., 2020; Strobbe et al., 2013; Tartaglia et al., 2015; Costagliola et al., 2014; Di Nardo et al., 2024), oral capsule (Giammusso et al., 2017)), PEA dosage (400 mg/bid (Cobellis et al., 2011), 200 mg/day (Cremon et al., 2017), 600 mg/day (Gagliano et al., 2011; Rossi et al., 2020), 300 mg/day (Giammusso et al., 2017), 400 gm/day (Murina et al., 2013), 300 mg/bid (Strobbe et al., 2013; Costagliola et al., 2014), 200 mg/tid (Di Nardo et al., 2024)), PEA period of exposure (12 weeks (Cobellis et al., 2011; Cremon et al., 2017; Giammusso et al., 2017; Strobbe et al., 2013; Di Nardo et al., 2024), 8 weeks (Gagliano et al., 2011; Murina et al., 2013), 16 weeks (Rossi et al., 2020), 10 days (Tartaglia et al., 2015), 24 weeks (Costagliola et al., 2014)), PEA use as monotherapy (Gagliano et al., 2011; Cobellis et al., 2011; Cremon et al., 2017; Giammusso et al., 2017; Strobbe et al., 2013; Tartaglia et al., 2015; Costagliola et al., 2014; Di Nardo et al., 2024) or add-on therapy (Murina et al., 2013; Rossi et al., 2020), level of concealment (double-blind (Gagliano et al., 2011; Cobellis et al., 2011; Cremon et al., 2017; Murina et al., 2013; Strobbe et al., 2013; Di Nardo et al., 2024), single-blind (Giammusso et al., 2017; Rossi et al., 2020), open-label (Costagliola et al., 2014)), and comparison to placebo (Gagliano et al., 2011; Cobellis et al., 2011; Cremon et al., 2017; Murina et al., 2013; Strobbe et al., 2013; Tartaglia et al., 2015; Di Nardo et al., 2024) or control treatments (Giammusso et al., 2017; Rossi et al., 2020; Costagliola et al., 2014). Seven studies systematically reported no PEA-related AEs during the observation period (Gagliano et al., 2011; Cobellis et al., 2011; Cremon et al., 2017; Giammusso et al., 2017; Rossi et al., 2020; Strobbe et al., 2013; Tartaglia et al., 2015; Costagliola et al., 2014; Di Nardo et al., 2024).

#### 3.5.1 Effect of Palmitoylethanolamide supplementation on gynecological and genitourinary pain or altered function

Two RCTs exploring gynecological disturbances demonstrated greater improvements of dysmenorrhea (Cobellis et al., 2011) and pelvic pain (Cobellis et al., 2011; Tartaglia et al., 2015) among PEA-treated EMS (Cobellis et al., 2011) and primary dysmenorrhea (Tartaglia et al., 2015) patients compared to those on placebo, although indicating PEA inferiority to Cyclooxygenase-2 (COX-2) inhibitors (Cobellis et al., 2011). Opposedly, dyspareunia improved in EMS patients treated with PEA monotherapy (Cobellis et al., 2011), while no improvement was observed in patients with isolated vestibulodynia who received PEA as an add-on to transcutaneous electrical nerve stimulation (TENS) (Murina et al., 2013). A study examining PEA effects in CP/CPPS patients showed significant improvements on lower urinary tract symptoms, pain, voiding symptoms, and their impact on quality of life, but not over measures of erectile dysfunction, compared to treatment with Serenoa Repens (Giammusso et al., 2017).

#### 3.5.2 Effect of Palmitoylethanolamide supplementation on intraocular pressure and visual field

Four studies addressed this area, converging towards the evidence of a greater reduction of intraocular pressure among PEA-treated patients with POAG (Gagliano et al., 2011; Rossi et al., 2020), NTG (Rossi et al., 2020; Costagliola et al., 2014), and isolated OH (Strobbe et al., 2013), compared to those treated with placebo (Gagliano et al., 2011; Strobbe et al., 2013), topical therapy alone (Rossi et al., 2020), or untreated (Costagliola et al., 2014). PEA was shown to significantly reduce IOP values among PEA-treated versus comparator-treated patients across all studies (Gagliano et al., 2011; Rossi et al., 2020; Strobbe et al., 2013; Costagliola et al., 2014), alongside improvements on the P50 component of pattern electroretinogram (PERG) (Rossi et al., 2020), peripheral endothelial dysfunction (Strobbe et al., 2013), and quality of life scores among POAG/NTG patients (Rossi et al., 2020). Contrasting results emerged regarding PEA ability to ameliorate measures of visual field (Gagliano et al., 2011; Rossi et al., 2020; Costagliola et al., 2014) and visual acuity (Gagliano et al., 2011; Costagliola et al., 2014). PEA did not significantly impact measures of central corneal thickness (Gagliano et al., 2011), cup-to-disk (C/D) ratio (Gagliano et al., 2011), and other PERG components (Rossi et al., 2020).

#### 3.5.3 Effect of Palmitoylethanolamide supplementation on gastrointestinal pain or altered function

Two RCTs documented greater improvements of abdominal pain/discomfort among PEA-treated IBS adult (Cremon et al., 2017) and pediatric (Di Nardo et al., 2024) patients compared to those treated with placebo, alongside significantly higher responder rates across both populations (Cremon et al., 2017; Di Nardo et al., 2024). Only among children, PEA was superior to placebo in reducing the frequency of episodes of abdominal pain and scores of life interference (Di Nardo et al., 2024). PEA did not significantly impact measures of epigastric pain frequency (Cremon et al., 2017), bloating frequency (Cremon et al., 2017), episodes of vomiting frequency (Cremon et al., 2017), as well as measures of bowel habit (Cremon et al., 2017; Di Nardo et al., 2024) and stool form (Cremon et al., 2017).

### 3.6 Effect of Palmitoylethanolamide supplementation on blood/plasma or other tissue biomarkers alterations across human conditions

Overall, ten studies addressed this area, focusing on PEA-dependent modulation of blood/plasma (Orefice et al., 2016; Pickering et al., 2022; Rao et al., 2021; Steels et al., 2019; Briskey et al., 2023; Kadanangode et al., 2023; Cobellis et al., 2011; Cremon et al., 2017; Albanese et al., 2022) or other tissue (e.g., gingival crevicular fluid) (Isola et al., 2021) inflammatory and oxidative stress markers (Orefice et al., 2016; Pickering et al., 2022; Isola et al., 2021; Briskey et al., 2023; Kadanangode et al., 2023; Cobellis et al., 2011; Cremon et al., 2017; Albanese et al., 2022), pathology-unspecific and -specific hematology or biochemistry markers (Pickering et al., 2022; Rao et al., 2021; Steels et al., 2019; Albanese et al., 2022), and eCBs/NAEs signaling (Orefice et al., 2016; Cremon et al., 2017). Across RCTs investigating PEA effects in β1a-IFN-treated RR-MS (Orefice et al., 2016), diabetes (Pickering et al., 2022), SRP-treated periodontitis (Isola et al., 2021), AR (Briskey et al., 2023), arthritis (Kadanangode et al., 2023), EMS (Cobellis et al., 2011), IBS (Cremon et al., 2017), and COVID-19 (Albanese et al., 2022) compared to placebo, standard treatment, or baseline condition, PEA intake led to significantly adjusted levels of several inflammatory biomarkers, including decreased Interleukin-1β (IL-1β) (Isola et al., 2021; Kadanangode et al., 2023), decreased Interleukin-6 (IL-6) (Pickering et al., 2022; Kadanangode et al., 2023; Albanese et al., 2022), decreased Interferon Gamma (IFN-γ) (Orefice et al., 2016), decreased Interleukin-17 (IL-17) (Orefice et al., 2016), decreased Interleukin-8 (IL-8) (Briskey et al., 2023), decreased Interleukin-4 (IL-4) (Briskey et al., 2023), decreased histamine (Briskey et al., 2023), decreased IL-1β/IL-10 ratio (Isola et al., 2021), decreased D-Dimer (Albanese et al., 2022), decreased Cluster of differentiation 3 (CD3^+^) and Cluster of differentiation 8 (CD8^+^) lymphocytes absolute count (Albanese et al., 2022), decreased Anti-SARS-CoV-2 Immunoglobulins G (IgG) (Albanese et al., 2022), increased Lymphocytes count (Albanese et al., 2022), decreased Neutrophil/Lymphocytes ratio (Albanese et al., 2022), decreased free oxygen radicals (Albanese et al., 2022), and at least a tendency to decreased Tumor Necrosis Factor-Alpha (TNF-α) (Orefice et al., 2016; Isola et al., 2021; Briskey et al., 2023; Kadanangode et al., 2023; Albanese et al., 2022). Contrasting results emerged regarding PEA effects on modulating levels of C-Reactive Protein (CRP), either significantly decreasing (Pickering et al., 2022; Albanese et al., 2022) or not (Cobellis et al., 2011). Also contrasting results emerged regarding PEA effects on modulating levels of Interleukin-10 (IL-10), either significantly increasing (Isola et al., 2021) or decreasing (Briskey et al., 2023). PEA showed no effects on Erythrocyte Sedimentation Rate (ESR) blood levels in EMS patients (Cobellis et al., 2011), immune cell and mastocyte activation in IBS patients (Cremon et al., 2017), and fibrinogen in both diabetic (Pickering et al., 2022) and COVID-19 patients (Albanese et al., 2022).

All pathology-unspecific hematology and biochemistry tests remained within normal ranges throughout the studies among PEA-treated DSP (Rao et al., 2021), knee OA (Steels et al., 2019), and COVID-19 (Albanese et al., 2022) patients.

Also, PEA treatment was not associated with changes in levels of fasting blood glucose and glycated hemoglobin among diabetic patients (Pickering et al., 2022).

Circulating eCBs/NAEs and related enzymes were modulated by PEA intake compared to placebo among β1a-IFN-treated RR-MS patients, including significant increase in PEA, AEA, and OEA plasma levels, lower expression of FAAH, and an inverse correlation between PEA and cytokines plasma levels (Orefice et al., 2016). On the contrary, no impact on eCBs/NAEs levels was observed among PEA-treated IBS patients (Cremon et al., 2017).

## 4 Discussion

Existing reviews interrogating the therapeutic role of PEA encompassed various designs of clinical studies and focused on specific symptoms or PEA formulations (Lang-Illievich et al., 2023; Capra et al., 2023; Santonocito et al., 2023; Scuteri et al., 2022; Indraccolo et al., 2022; Artukoglu et al., 2017; Schweiger et al., 2024). However, to the best of our knowledge, this is the first systematic review collecting all RCTs examining the effects of PEA supplementation across human illnesses, aiming to comprehensively address its impact on any targeted symptoms, either measured clinically or using appropriate assessment scales.

This systematic review indicates several prominent treatment implications of PEA supplementation that deserve to be highlighted.

Not surprisingly, the most convincing findings emerged from RCTs examining PEA efficacy on pain severity across several clinical conditions, encompassing neuropathic pain (Andresen et al., 2016; Evangelista et al., 2018; Pickering et al., 2022; Coraci et al., 2018; Faig-Marti and Martinez-Catassus, 2017; Faig-Marti and Martinez-Catassus, 2020; Guida et al., 2010; Ottaviani et al., 2019), migraine-associated pain (Briskey et al., 2024), somatic pain (Orefice et al., 2016; Steels et al., 2019; Bacci et al., 2011; Germini et al., 2017; Isola et al., 2021; Marini et al., 2012; Kadanangode et al., 2023), visceral pain (Cobellis et al., 2011; Cremon et al., 2017; Giammusso et al., 2017; Tartaglia et al., 2015; Di Nardo et al., 2024), and nociplastic pain (Salaffi et al., 2023). Evidence largely pointed to improved unidimensional [e.g., Numeric Rating Scale (NRS), Visual Analogue Scale (VAS)] (Evangelista et al., 2018; Orefice et al., 2016; Pickering et al., 2022; Steels et al., 2019; Guida et al., 2010; Ottaviani et al., 2019; Briskey et al., 2024; Bacci et al., 2011; Germini et al., 2017; Isola et al., 2021; Marini et al., 2012; Salaffi et al., 2023; Cobellis et al., 2011; Tartaglia et al., 2015) and multidimensional [e.g., Patient Global Impression of Change (PGIC), Brief Pain Inventory-Diabetic Peripheral Neuropathy (BPI-DPN)] (Evangelista et al., 2018; Pickering et al., 2022; Steels et al., 2019; Faig-Marti and Martinez-Catassus, 2017, 2020; Kadanangode et al., 2023; Cobellis et al., 2011; Cremon et al., 2017; Giammusso et al., 2017; Di Nardo et al., 2024) pain measures, also underscoring PEA effect on daily functioning (Orefice et al., 2016; Pickering et al., 2022; Steels et al., 2019; Guida et al., 2010; Salaffi et al., 2023), as a result of pain management. When PEA did not significantly impact pain intensity scores, recovered sensory conduction and morphology measures (Coraci et al., 2018) or a reduction in the use of conventional pain medications (Andresen et al., 2016) were at least observed. In the few cases when the nature of the non-placebo comparator was clearly defined, PEA showed its superiority to both NSAIDs (Marini et al., 2012; Kadanangode et al., 2023) and other natural products with anti-inflammatory properties (i.e., Serenoa Repens) (Giammusso et al., 2017), but not to COX-2 inhibitors (Cobellis et al., 2011). Recent robust systematic reviews and metanalyses on this topic have already yielded superior pooled estimates of PEA analgesic effect over comparators, particularly in the treatment of chronic pain (Lang-Illievich et al., 2023; Scuteri et al., 2022; Schweiger et al., 2024), with favorable outcomes on quality of life (Lang-Illievich et al., 2023), sleep quality (Lang-Illievich et al., 2023), and physical function (Lang-Illievich et al., 2023), as well as no notable side effects (Lang-Illievich et al., 2023), although the high methodological heterogeneity (e.g., dosing regimen, PEA formulations) and small primary samples size may have limited the statistical power of the conclusions drawn (Lang-Illievich et al., 2023; Scuteri et al., 2022; Schweiger et al., 2024). Additionally, our systematic reappraisal unveiled a paucity of RCTs showing beneficial effects of PEA in the treatment of neurological motor symptoms occasionally but not exclusively associated to neuropathic pain, including reduced mobility, spasticity, stiffness, and increased fatigability (Andresen et al., 2016; Campolo et al., 2021; Orefice et al., 2016). A more in-depth investigation of this area through RCTs may be relevant, given the evidence of PEA benefits on neuromotor symptoms (Bonzanino et al., 2024; Palma et al., 2016; Brotini et al., 2017) and muscle recovery (Palma et al., 2016; Mallard et al., 2020) both in clinical (Bonzanino et al., 2024; Palma et al., 2016; Brotini et al., 2017) and healthy (Mallard et al., 2020) populations. Noteworthy, among PEA-treated diabetic (Pickering et al., 2022), RR-MS (Orefice et al., 2016), periodontitis (Isola et al., 2021), and arthritis (Kadanangode et al., 2023) patients, both pain improvement (Orefice et al., 2016; Pickering et al., 2022; Isola et al., 2021; Kadanangode et al., 2023) and better general wellbeing (e.g., cognitive function, sleep quality) (Orefice et al., 2016; Pickering et al., 2022) were accompanied by contextual peripheral tissues lowering proinflammatory biomarkers (e.g., TNF-α, IL-1β, CRP, IL-6, IFN-γ, IL-17) (Orefice et al., 2016; Pickering et al., 2022; Isola et al., 2021; Kadanangode et al., 2023), increasing anti-inflammatory biomarkers (e.g., IL-10) (Isola et al., 2021), and enhanced eCBs/NAEs signaling (e.g., lower FAAH expression, increased PEA, AEA, and OEA plasma levels) (Orefice et al., 2016), indicating that PEA exerts its effects over factors either precociously altered in the early phases of a disease (Orefice et al., 2016), or contributing to illness progression (Pickering et al., 2022; Isola et al., 2021). Few other studies explored potential correlations between pain relief and blood/plasma biomarkers modulation, failing to retrieve significant results, and suggesting the possibility that PEA may act through different pathways depending on the clinical condition (Steels et al., 2019; Cobellis et al., 2011; Cremon et al., 2017), with the potential for long-lasting effects even after treatment discontinuation (e.g., through TRPV1 desensitization) (Cremon et al., 2017). Indeed, PEA ability to modulate immune responses and halt the release of inflammatory mediators, driven by its diverse molecular and cellular interactions, appears to be the most accredited mechanism underlying its efficacy in controlling chronic pain, where neuroinflammation contributes to the persistence of pain signals (Scuteri et al., 2022; Varrassi et al., 2024). However, correlation between pain management and inflammatory biomarkers or eCBs/NAEs signaling modulation as a response to PEA treatment remains largely unexplored across RCTs, warranting the need of further studies to fill this literature gap.

Numerous studies have investigated PEA effects on neuropsychiatric symptoms, either as primary (Abedini et al., 2022; Campolo et al., 2021; Evangelista et al., 2018; Ghazizadeh-Hashemi et al., 2018; Khalaj et al., 2018; Lunardelli et al., 2019; Rao et al., 2021; Salehi et al., 2022; Versace et al., 2023) or secondary (Andresen et al., 2016; Gagliano et al., 2011; Orefice et al., 2016; Pickering et al., 2022; Steels et al., 2019; Bonzanino et al., 2024) objectives, showing a general trend towards improved wellbeing in PEA-treated patients. First, depressive and acute manic symptoms appeared to be positively affected by PEA add-on to conventional therapy in MDD (Ghazizadeh-Hashemi et al., 2018) and BPAD (Abedini et al., 2022) patients respectively, possibly thanks to PEA multi-faceted mechanism of action, including PPAR-α-mediated anti-inflammatory effects, GPR55 agonism (with implications for the modulation of monoaminergic neurons), TRPV1 allosteric modulation, glutamate neuro-toxicity prevention, and neurogenesis promotion (Petrosino and Schiano Moriello, 2020; Petrosino and Di Marzo, 2017). Improved depressive symptoms were also observed among PEA-treated diabetic patients (Pickering et al., 2022), being less clear across other conditions only secondarily affecting mood (Andresen et al., 2016; Campolo et al., 2021; Steels et al., 2019). Second, most RCTs investigating PEA short- (Lunardelli et al., 2019) or long-term (Campolo et al., 2021; Orefice et al., 2016; Versace et al., 2023; Bonzanino et al., 2024) therapeutic potential in treating conditions associated with impaired cognitive function have yielded solid favorable results, either using psychometric measures (Campolo et al., 2021; Lunardelli et al., 2019; Bonzanino et al., 2024), or subjective reports (Orefice et al., 2016), or neurophysiological assessments (Versace et al., 2023), supposedly accounting for PEA ability to counteract neurodegenerative processes through the modulation of oxidative stress and neuroinflammation pathways, including astrocyte and microglia proliferation and neuronal loss (Colizzi et al., 2022). Intriguingly, PEA was also shown to exert its favorable effects on attention, executive function, and memory domains in healthy adults, possibly through increased neurotrophic factors signaling (Kim et al., 2024). Third, two out of three RCTs exploring PEA impact on sleep disturbances both as consequence and contributor of neuropathic pain pointed to PEA beneficial effects mostly on measures of sleep adequacy and sleep-related troubles on daily activities (Andresen et al., 2016; Evangelista et al., 2018; Pickering et al., 2022), whereas no significant improvements emerged from single studies assessing PEA effects on disrupted sleep secondary to somatic pain (Steels et al., 2019) and on primary DSP (Rao et al., 2021). Fourth, PEA add-on to second-generation antipsychotics exerted beneficial effects on both residual negative psychotic symptoms in SCZ (Salehi et al., 2022) and behavioral and speech disturbances in ASD (Khalaj et al., 2018), underscoring its potential impact on neurodevelopmental manifestations driven by mesocorticolimbic dopaminergic disruption and glutamate neurotoxicity (Colizzi et al., 2021; Bortoletto et al., 2023), sustained by inflammatory processes (Dunleavy et al., 2022; Masi et al., 2015; Arteaga-Henriquez et al., 2022), and often difficult to treat or unresponsive to conventional psychotropic medications (Tseng et al., 2022; Krause et al., 2018; Williams et al., 2013; Hurwitz et al., 2012).

Alongside these two main strains of research, additional evidence appears more scattered but is still worth mentioning. First, narrow but methodologically consistent data emerged from five RCTs exploring Co-ultraPEALut in combination to olfactory training on chronic hyposmia/anosmia recovery and, to a lesser extent, parosmia recovery in COVID-19 patients, possibly reducing lingering neuroinflammation of the olfactory bulb and higher olfactory centers following SARS-CoV-2 infection (D'Ascanio et al., 2021; Di Stadio et al., 2022; Di Stadio et al., 2023a; Cantone et al., 2024; Di Stadio et al., 2023b; Capra et al., 2023). These findings are also coherent with the previously discussed beneficial effects of PEA on brain fog and cognitive fatigue in the context of long-COVID syndrome (Versace et al., 2023), which further highlight the anti-neuroinflammatory potential of PEA. Second, aligning with recent meta-analysis (Gagliano et al., 2011; Strobbe et al., 2013; Costagliola et al., 2014; Crupi et al., 2024), PEA was shown to significantly reduce IOP among glaucoma patients across four RCTs (Gagliano et al., 2011; Rossi et al., 2020; Strobbe et al., 2013; Costagliola et al., 2014), due to still unclear mechanisms that may include the modulation of peripheral endothelial dysfunction (Strobbe et al., 2013). Finally, two RCTs converge towards the evidence of PEA efficacy in the context of allergic symptoms, both respiratory (Briskey et al., 2023) and cutaneous (Rao et al., 2023b), possibly manifesting its effects through the reduction of histamine and multiple immune biomarkers levels.

In addition to its primary therapeutic effects, the considerations outlined above highlight how PEA supplementation may serve as a valuable treatment for transdiagnostic symptoms intertwining neuropsychiatric and physical conditions, especially those fueled by neuroinflammatory processes—both in the short and in the long term. This aspect would be particularly noticeable in the case of formulations with smaller particle size (Andresen et al., 2016; Campolo et al., 2021; Evangelista et al., 2018; Ghazizadeh-Hashemi et al., 2018; Lunardelli et al., 2019; Orefice et al., 2016; Versace et al., 2023; Bonzanino et al., 2024; D'Ascanio et al., 2021; Di Stadio et al., 2022; Di Stadio et al., 2023a; Guida et al., 2010; Ottaviani et al., 2019; Palma et al., 2016; Cantone et al., 2024; Di Stadio et al., 2023b; Bacci et al., 2011; Germini et al., 2017; Cobellis et al., 2011; Cremon et al., 2017; Rossi et al., 2020; Costagliola et al., 2014; Di Nardo et al., 2024; Albanese et al., 2022) or those produced using cold-water dispersible technology (Pickering et al., 2022; Rao et al., 2021, 2023a, 2023b; Steels et al., 2019; Briskey et al., 2023, 2024), maximizing PEA bioavailability and efficacy. To this extent, it is noteworthy that most RCTs addressing PEA effects on neuropsychiatric or neurologic disturbances involved micronized/ultramicronized formulations, either alone (Andresen et al., 2016; Evangelista et al., 2018; Ghazizadeh-Hashemi et al., 2018; Orefice et al., 2016; Guida et al., 2010; Ottaviani et al., 2019; Palma et al., 2016) or in combination with flavonoids or other antioxidant compounds (e.g., luteolin, ALA) (Campolo et al., 2021; Lunardelli et al., 2019; Versace et al., 2023; Bonzanino et al., 2024; D'Ascanio et al., 2021; Di Stadio et al., 2022; Di Stadio et al., 2023a; Cantone et al., 2024; Di Stadio et al., 2023b), or LipiSperse PEA (Pickering et al., 2022; Rao et al., 2021; Steels et al., 2019; Briskey et al., 2024), possibly to favor PEA penetration into the nervous system through the blood-brain barrier (Petrosino and Di Marzo, 2017; Petrosino et al., 2016; Briskey et al., 2019).

Moreover, PEA safety and tolerability would make it an excellent and acceptable option for individuals already receiving pharmacological treatments for their underlying condition, either during cross-titration if the current medication is being gradually discontinued, or as an add-on therapy when additional support is needed, or in the management of unpleasant side effects (Orefice et al., 2016).

### 4.1 Limitations and literature gaps

The findings emerging from our systematic review should also be seen considering some limitations, regarding either the characteristics of included studies or the methodological approach adopted. First, despite our work focused on clinical populations, only few studies selected were conducted in large samples (>100 patients) (Rao et al., 2021; Steels et al., 2019; Di Stadio et al., 2022; Di Stadio et al., 2023a; Guida et al., 2010; Di Stadio et al., 2023b; Masek et al., 1974; Briskey et al., 2023; Rao et al., 2023a; Salaffi et al., 2023; Tartaglia et al., 2015) or at multiple sites (Abedini et al., 2022; Andresen et al., 2016; Ghazizadeh-Hashemi et al., 2018; Khalaj et al., 2018; Salehi et al., 2022; Di Stadio et al., 2022; Guida et al., 2010; Masek et al., 1974; Cremon et al., 2017), with the majority carried out in Italy (Campolo et al., 2021; Evangelista et al., 2018; Gagliano et al., 2011; Lunardelli et al., 2019; Orefice et al., 2016; Versace et al., 2023; Bonzanino et al., 2024; Coraci et al., 2018; D'Ascanio et al., 2021; Di Stadio et al., 2022; Di Stadio et al., 2023a; Guida et al., 2010; Ottaviani et al., 2019; Palma et al., 2016; Cantone et al., 2024; Di Stadio et al., 2023b; Bacci et al., 2011; Germini et al., 2017; Isola et al., 2021; Marini et al., 2012; Salaffi et al., 2023; Cobellis et al., 2011; Cremon et al., 2017; Giammusso et al., 2017; Murina et al., 2013; Rossi et al., 2020; Strobbe et al., 2013; Tartaglia et al., 2015; Costagliola et al., 2014; Di Nardo et al., 2024; Albanese et al., 2022). This may result in some differences between the study participants and the population typically treated in routine clinical practice, alongside the frequent inclusion of otherwise healthy patients without any comorbid physical, neurological, or mental health conditions (see Supplementary Table). To improve the interpretation and generalizability of the findings about PEA supplementation across all clinical conditions, larger multicenter RCTs will be necessary.

Second, despite compelling, converging evidence on the treatment of neuropsychiatric symptoms is still limited, particularly from RCTs investigating PEA effects in populations with primary neuropsychiatric disturbances, suggesting interpreting presented findings with caution. Indeed, adjuvant therapy with PEA showed to enhance the effectiveness of ongoing psychotropic medications in overt mental health conditions, possibly allowing a reduction in the necessary dosage of treatments that are not devoid of severe adverse effects (Abedini et al., 2022; Ghazizadeh-Hashemi et al., 2018; Khalaj et al., 2018; Salehi et al., 2022). However, there is still lack of RCTs exploring PEA as monotherapy for conditions that are often treatment-orphan (e.g., first-episode seizures, depressive symptoms in ASD, and clinical high-risk states for psychosis), particularly in the early stages preceding the onset of full-blown neuropsychiatric disorders or during remission phases, with potential disease-modifying properties. Moreover, evidence linking improvements in neuropsychiatric symptoms with inflammatory response or eCBs/AEs signaling is still scarce (Orefice et al., 2016), and it remains unclear whether PEA effects correlate with the modulation of other biological systems. Future studies across all conditions will be needed to systematically investigate the correlation between clinical response to PEA supplementation and the modulation of biological markers, potentially considering its direct impact on microbiome composition and function as well (Batacan et al., 2024; Brankovic et al., 2024; Pirozzi et al., 2023; Minichino et al., 2021).

Third, while the use of non-placebo comparators was consistently established across the studies (Campolo et al., 2021; Evangelista et al., 2018; Lunardelli et al., 2019; Bonzanino et al., 2024; D'Ascanio et al., 2021; Palma et al., 2016; Bacci et al., 2011; Isola et al., 2021; Marini et al., 2012; Yuan et al., 2014; Kadanangode et al., 2023; Rao et al., 2023b; Salaffi et al., 2023; Cobellis et al., 2011; Giammusso et al., 2017; Costagliola et al., 2014; Albanese et al., 2022), in most cases its nature was not explicitly defined, instead described with terms like “standard therapy” or “standard treatment”, thereby limiting comparisons with other renowned anti-inflammatory medications (Marini et al., 2012; Kadanangode et al., 2023; Cobellis et al., 2011; Giammusso et al., 2017). Also, only a limited number of studies have compared different PEA formulations (Cantone et al., 2024) and dosages (Evangelista et al., 2018; Di Stadio et al., 2023b), unveiling a need for further investigation before definitive conclusions can be drawn regarding PEA efficacy for the treatment of certain disorders. To this end, although most studies were rated as high quality using the JBI critical appraisal for RCTs (Barker et al., 2023), this tool fails to capture specific details on the trial medication, which were omitted in a few studies (Abedini et al., 2022; Gagliano et al., 2011; Khalaj et al., 2018; Salehi et al., 2022; Coraci et al., 2018; Faig-Marti and Martinez-Catassus, 2017, 2020; Marini et al., 2012; Strobbe et al., 2013). This information, along with different dosages, intake routes, period of exposure, and use as monotherapy or add-on treatment and is crucial for understanding PEA effects and accurately comparing results and side effects across studies.

Finally, although we searched three major medical databases, reviewed two clinical trial registries, and manually checked all reference lists, some RCTs may still have been missed, a limitation inherent to all systematic reviews.

## 5 Conclusion

This systematic review sought to provide a comprehensive overview of the therapeutic potential of PEA across all RCTs exploring its efficacy and safety in clinical populations. While further research is necessary, accumulating literature has already underscored PEA as a versatile dietary supplement with a favorable safety profile for both long- and short-term treatment of several transdiagnostic symptoms, offering potential disease-modifying effects and a biological profile closely aligned with human physiology.

## CRediT authorship contribution statement

**R. Bortoletto:** Writing – review & editing, Writing – original draft, Software, Methodology, Investigation, Data curation, Conceptualization. **C. Comacchio:** Writing – review & editing, Methodology, Investigation, Data curation, Conceptualization. **M. Garzitto:** Writing – review & editing, Methodology, Data curation, Conceptualization. **F. Piscitelli:** Writing – review & editing, Methodology, Data curation, Conceptualization. **M. Balestrieri:** Writing – review & editing, Methodology, Data curation, Conceptualization. **M. Colizzi:** Writing – review & editing, Writing – original draft, Supervision, Methodology, Data curation, Conceptualization.

## Funding

This work received no external funding.

## Declaration of competing interest

MC has been a consultant/advisor to GW Pharma Limited, F. Hoffmann-La Roche Limited, and GW Pharma Italy SRL, outside of this work. The remaining authors declare that the research was conducted in the absence of any commercial or financial relationships that could be construed as a potential conflict of interest.

## Data Availability

No data was used for the research described in the article.
