# Supplementary material for: Palmitoylethanolamide supplementation for human health: A state-of-the-art systematic review of Randomized Controlled Trials in patient populations
Source: Brain Behav Immun Health. 2024 Dec 23;43:100927. doi: 10.1016/j.bbih.2024.100927 (PMC11745966; doi:10.1016/j.bbih.2024.100927)
Supplement: Multimedia component 1 [file mmc1.docx]

Supplementary Table 1. Eligibility criteria of Randomized Controlled Trials investigating Palmitoylethanolamide supplementation across human diseases.

| **Study ID** | **Inclusion criteria** | **Exclusion criteria** |
| --- | --- | --- |
| Abedini et al., 2022 (Iran) (41) | 1. Inpatients  2. 18 to 54 years of age  3. Moderate to severe manic episode (YMRS ≥ 20) | 1. IQ < 70  2. Allergy to lth, rsp, or PEA  3. Substance dependence (except nicotine and caffeine)  4. Ongoing manic-inducing medications  5. Current metabolic disorders  6. Current severe hepatic disease |
| Albanese et al., 2022 (Italy) (88) | 1. 18 to 80 years of age  2. Past 24 h confirmed virological COVID-19 with no symptoms or mild-to-moderate infection | 1. Pregnant or breastfeeding women  2. Severe respiratory failure requiring invasive mechanic ventilation  3. Allergy or hypersensitivity to PEA or to ≥ 1 of its excipients |
| Andresen et al., 2016 (Denmark, Norway, United Kingdom) (42) | 1. ≥ 18 years of age  2. ≥ 6 month-lasting SCI  3. ≥ 3 month-lasting at- and/or below-level SCI neuropathic pain  4. 4 ≤ average pain intensity ≤ 9, during a 7-day baseline period | 1. Concomitant severe cerebral damage, terminal illness  2. Planned surgery  3. Alcohol, or substance abuse  4. Hypersensitivity to umPEA or its excipients  5. Current psychiatric disease (except reactive depression)  6. Pregnant or breastfeeding women, insufficient contraceptives  7. Impossibility to differentiate neuropathic SCI pain  from other types of pain |
| Bacci et al., 2011 (Italy) (68) | 1. 18 to 30 years of age  2. No signs of inflammation or tooth decay  3. Lower third molars identical in position, orientation, depth, and class of impaction | 1. Patients needing monolateral extractions  2. Past 2 months other clinical studies involvement  3. Systemic diseases and taking chronic pharmacotherapy  4. Unlikelihood of compliance  5. Allergies to NSAIDs  6. Impossibility to take NSAIDs |
| Bonzanino et al., 2024 (Italy) (55) | 1. ≥ 18 and ≤ 90 years of age  2. 5 ≤ NIHSS score ≤ 20  3. No significant pre-stroke disabilities | 1. Hemorrhagic stroke  2. Concomitant diseases interfering with evaluation |
| Briskey et al., 2023 (Australia) (74) | 1. ≥ 18 years of age  2. Good general health  3. Providing written informed consent  4. Experiencing seasonal allergic rhinitis | 1. Using any allergy relief supplements or products during the study  2. Pregnant or breastfeeding women, insufficient contraceptives |
| Briskey et al., 2024 (Australia) (67) | 1. ≥ 18 years of age  2. Good general health  3. At least five attacks fulfilling ICHD3 criteria B-D  4. Headache attacks lasting 4–72 h (untreated or unsuccessfully treated)  5. Headaches having ≥ 2/4 following: (a) unilateral location, (b) pulsating quality, (c) moderate or severe pain intensity, (d) aggravated by or causing avoidance of routine physical activity  6. During headaches, ≥ 1 following: (a) nausea and/or vomiting, (b) photophobia, (c) phonophobia  7. Migraine not better accounted for by another ICHD3 diagnosis | 1. Chronic past and/or current alcohol use (>14 alcoholic drinks per  week)  2. Using long-term medication (unless for controlled medical  conditions as above)  3. Pregnant or breastfeeding women, insufficient contraceptives  4. Allergic, or hypersensitive to any of the ingredients in  the active or placebo formula  5. Used preventative medication  6. Migraines to occur on ≥ 15 days of the month for > 3 months, which, on ≥ 8 days per month, had the features of a migraine headache, a debilitating attack lasting > 72 h, or a seizure |
| Campolo et al., 2021 (Italy) (43) | 1. All consecutive admitted patients  2. Moderate TBI (GCS 9-13) | X |
| Cantone et al., 2024 (Italy) (65) | 1. 18 to 60 years of age  2. Confirmed history of COVID-19 (positive nasopharyngeal swab for SARS-CoV-2)  3. Smell disturbances resulting from the COVID-19 episode  4. Persistent olfactory disfunction at time of recruitment (smell loss and parosmia) | 1. Known neuroinflammatory or neurodegenerative diseases  2. Stroke or head trauma within the past 5 years  3. Prior or current chemo-radiotherapy  4. Prior or current sinonasal cancer or chronic rhinosinusitis  5. Prior or current nasal allergy  6. Prior or current nasal polyps  7. Anatomical conditions reducing nasal airflow  8. Ongoing medications interacting with olfactory function  9. Current severe psychiatric disorders. |
| Cobellis et al., 2011 (Italy) (79) | EMS diagnosis | X |
| Coraci et al., 2018 (Italy) (56) | 1. Minimum/mild idiopathic CTS (Padua’s classification)  2. Providing written informed consent | 1. Other neuropathies  2. Orthopedic, endocrinological and rheumatological diseases |
| Costagliola et al., 2014 (Italy) (86) | 1. NTG diagnosis (optic disc abnormalities consistent with glaucomatous optic neuropathy with visual field loss)  2. IOP ≤ 18 mmHg without topical hypotensive therapy  3. ≥ 18 years of age  4. logMAR BCVA ≥ 0.70  5. Progression of anatomic and functional damage as determined by computerized perimetry and serial stereophotographs of the optic nerve head  6. No history of amblyopia  7. No history of ocular disease or previous eye surgery | 1. Active ocular disease  2. Use of other ocular medications or therapies that might have  a substantial effect on IOP  3. History of ocular surgery  4. Use of other similar systemic medications  5. Vasoactive systemic therapies (e.g., Ca-antagonists,  oral beta-blocker, etc.)  6. Current tobacco smoker  7. Pregnant or breastfeeding women |
| Cremon et al., 2016 (Italy, Spain, France, Croatia, Bosnia) (80) | 1. All IBS subtypes (IBS-C, IBS-D, IBS-M)  2. 18 to 70 years of age  3. Previous 5 years negative colonoscopy or barium enema examination  4. Negative relevant additional screening or consultation | 1. Pregnant or breastfeeding women, insufficient contraceptives  2. Current use of NSAIDs, corticosteroids, and mast cell stabilizers  3. Past month use of topical or systemic antibiotics  4. Continuous use of stimulant laxatives  5. Major abdominal surgery  6. Medical history, appropriate consultations, and laboratory tests  confirmation of IBD, infectious diarrhoea or diverticular disease,  coeliac disease, allergic diseases (including asthma),  other organic or psychiatric disorders  7. Impossibility to take NSAIDs |
| D’Ascanio et al., 2021 (Italy) (57) | 1. Outpatients  2. 18 to 90 years of age  3. Confirmed history of COVID-19 (positive nasopharyngeal swab for SARS-CoV-2)  4. Confirmed anosmia/hyposmia (Sniffin’ Sticks psychophysical test, TDI < 31) persisting ≥ 90 days after subsequent negative COVID-19 nasopharyngeal swab | 1. History of olfactory-gustatory disorders  2. Impaired cognitive function  3. History of NDD (AD and PDis)  4. Medical therapy with known detrimental effects on olfactory  function  5. Active rhinological disorders  6. History or chemo-radiotherapy of the head/neck region  7. History of stroke or neurotrauma  8. Severe nasal blockage from stenosis or deformity  9. Severe psychiatric illness  10. Previous sinonasal or nasopharyngeal tumors |
| Di Nardo et al., 2024 (Italy) (87) | 1. 10 to 17 years of age  2. All IBS subtypes (IBS-C, IBS-D, IBS-M)  3. Negative findings for fecal calprotectin and anti-transglutaminase antibodies | 1. Current use of NSAIDs, corticosteroids, and mast cells stabilizers  2. Past month use of topical or systemic antibiotics  3. Continuous use of stimulant laxatives  4. Major abdominal surgery  5. Medical history, appropriate consultations, and laboratory tests  confirmation of IBD, infectious diarrhoea, allergic diseases,  other organic or psychiatric disorders |
| Di Stadio et al., 2022 (Italy) (58) | 1. Outpatients  2. 18 to 65 years of age  3. ≥ 180 days olfactory impairment post negative COVID-19 nasopharyngeal swab | 1. History of olfactory-gustatory disorders  2. Active chemotherapy or estrogen inhibitors-therapy (aromatase)  3. Impaired cognitive function  4. History of NDD  5. Medical therapy with detrimental effects on olfactory function  6. Active rhinological disorders  7. History or chemo-radiotherapy of the head/neck region  8. History of stroke or head trauma  9. Severe nasal blockage from stenosis or deformity  10. Severe psychiatric illness  11. History of sinonasal or nasopharyngeal tumors  12. Past 30 days corticosteroid therapy to treat olfactory dysfunction  13. Current anti-inflammatory or immunomodulant therapy |
| Di Stadio et al., 2023a (Italy) (59) | 1. Outpatients  2. 18 to 65 years of age  3. ≥ 180 days olfactory impairment post negative COVID-19 nasopharyngeal swab | 1. History of olfactory-gustatory disorders  2. Active chemotherapy or estrogen inhibitors-therapy (aromatase)  3. Impaired cognitive function  4. History of NDD  5. Active NDD/neuroinflammatory diseases  6. Medical therapy with detrimental effects on olfactory function  7. Active rhinological disorders  8. History or chemo-radiotherapy of the head and neck region  9. History of removal of sinonasal or nasopharyngeal tumors  10. < 3 years stroke or moderate/severe head trauma,  nasal septal deviation, or turbinate hypertrophy  11. Previous 30 days corticosteroid therapy to treat  olfactory dysfunction  12. Current anti-inflammatory or immunomodulant therapy |
| Di Stadio et al., 2023b (Italy) (66) | 1. Outpatients  2. 18 to 60 years of age  3. ≥ 180 days olfactory impairment post negative COVID-19 nasopharyngeal swab | 1. History of olfactory-gustatory disorders  2. Active chemotherapy or estrogen inhibitors-therapy (aromatase)  3. Impaired cognitive function  4. History of NDD  5. Medical therapy with detrimental effects on olfactory function  6. Active rhinological disorders  7. History or chemo-radiotherapy of the head and neck region  8. History of stroke or head trauma  9. Severe nasal blockage from stenosis or deformity  10. Severe psychiatric illness  11. History of sinonasal or nasopharyngeal tumors  12. Previous 30 days corticosteroid therapy to treat olfactory dysfunction  13. Current anti-inflammatory or immunomodulant therapy |
| Evangelista et al., 2018 (Italy) (44) | 1. ≥ 18 years of age  2. ≥ 6 months painful symptoms  3. Sleep disturbance due to nocturnal numbness and/or tingling and sustained arm or hand pain and mitigated by change in posture  4. PSQI score ≥ 8  5. 5 ≤ NRS ≤ 9  6. Never surgically treated unilateral CTS | 1. Concomitant clinical conditions interfering with  CTS painful symptoms evaluation  2. Previous surgically treated unilateral CTS |
| Faig-Marti and Martìnez-Catassùs, 2017  (Spain) (60)  Faig-Martì and Martìnez-Catassùs, 2020  (Spain) (61) | 1. 18 to 75 years of age  2. ≥ 3 months mild to moderate CTS (ENG) | 1. History of previous upper extremity surgery  2. Active treatment with steroids  3. Use of night splinting  4. Food allergies |
| Gagliano et al., 2011 (Italy) (45) | 1. ≥ 18 years of age  2. POAG/OH diagnosis  3. ≥ 3 months timolol 0.5% eyedrops twice daily  4. 19 mmHg < IOP < 24 mmHg  5. Able to perform a reliable visual field (a minimum of 3 tests)  6. < 0.6 C/D ratio | 1. Need for glaucoma surgical or laser therapy  2. Past year ocular surgery  3. No tolerability to product under evaluation  4. Visual acuity 8/10 with refractive error 3 diopters  5. Pupillary diameter 2.5 mm  6. Concomitant systemic or ocular pathologies  7. Pregnant or breastfeeding women  8. Vasoactive systemic therapies  9. Current tobacco smoker |
| Germini et al., 2017 (Italy) (69) | 1. ≥ 65 years of age  2. Back, joints, or limbs chronic pain for ≥ 6 months | 1. Chronic cancer pain  2. Subacute or chronic ischemic pain  3. Recently started new pharmacological or non-pharmacological  treatment for pain |
| Ghazizadeh-Hashemi et al., 2018 (Iran) (46) | 1. 18 to 50 years of age  2. HDRS Total ≥ 19; HDRS Item 1 ≥ 2 | 1. Past month antidepressant and psychotherapy  2. Past 2 months ECT  3. Psychosis or other mental disorders diagnosis  4. Alcohol or substance abuse or dependence (except nicotine)  5. Risk of suicide or suicidal ideation  6. Any uncontrolled medical problem |
| Giammusso et al., 2017  (Italy) (81) | 1. ≥ 6 months complaints of chronic pelvic pain  2. IPSS score > 13 at visit 1  3. Pain domain of NIH-CPSI > 1 at visit 1  4. Total PSA < 4 ng/ml  5. Sign and symptoms of category III CP/CPPS (NIH) | 1. Subjects < 22 and > 61 years of age  2. Major comorbidities  3. Known anatomical abnormalities of the urinary tract  4. Evidence of other urological diseases  5. Residual urine volume > 50 ml resulting from bladder outlet obstruction |
| Guida et al., 2010 (Italy) (62) | 1. 18 to 75 years of age  2. VAS ≥ 5 | 1. Unconfirmed lumbosciatalgia diagnosis at baseline  2. Pregnant women  3. Concomitant use of medications causing drug-induced  peripheral neuropathies  4. Comorbidities interfering with efficacy assessment  5. Past 4 weeks participation to a clinical study  6. Unlikelihood of compliance |
| Isola et al, 2021 (Italy) (70) | 1. Good general health  2. ≥ 6 teeth per quadrant  3. PD ≥ 4 mm and CAL ≥ 2 mm in ≥ 40% of the analyzed sites  4. ≥ 2 teeth in each quadrant with PD ≥ 5 mm  5. ≥ 40% sites with BOP  6. No furcation involvement | 1. Past 6 months periodontal therapy  2. Past 6 months use of antibiotics, anti-inflammatory, or  Immunosuppressant medication  3. Pregnant women  4. Any systemic condition which might affect the study  5. Past 3 months use of mouthwash containing antimicrobials  6. Use of hormonal contraceptives  7. Anti-inflammatory and immunosuppressive medications  8. History of excessive drinking  9. Current tobacco smoker  10. Class II and III tooth mobility |
| Kadanangode Narayanaswam et al., 2023 (India) (75) | 1. 18 to 60 years of age  2. DAS28 score ≥ 3  3. Providing written informed consent  4. Ability to comprehend the nature and objectives of the study and demonstrate a willingness to adhere to study procedures | 1. Current use of NSAIDs/painkillers other than standard drug  2. Abnormal results on liver function test  3. Current diabetic neuropathy  4. Current severe renal, hepatic, cardiac, gastrointestinal,  neurological, hematological, or respiratory disorder  5. Current psychiatric disorder  6. BMI > 35 kg/m^2^ or < 20 kg/m^2^  7. Need for surgery during the study period  8. Past 12-week participation in any clinical study or trial  9. Known hypersensitivity to the study drugs  10. Patients with severe infection  11. History of intake of any ayurvedic/herbal/homeopathic/dietary  supplements in the last two months  12. Pregnant or breastfeeding women, insufficient contraceptives |
| Khalaj et al., 2018 (Iran) (47) | 1. 4 to 12 years of age  2. Irritability symptoms of at least moderate severity (scores ≥ 12 on the ABC-C Irritability subscale) | 1. Symptoms not pronounced enough to be considered for  treatment with rsp  2. Concomitant psychiatric disorder  3. Preexisting medical condition  4. Severe intellectual disability  5. Alcohol/drug abuse  6. Dyskinesia  7. Past 6 months AP medication or behavior treatment |
| Lunardelli et al., 2019 (Italy) (48) | 1. > 75 years of age  2. Proximal hip fracture eligible for early surgery | 1. Severe dementia  2. Delirium at the time of admission  3. Patients receiving AP medications  4. Surgery cannot be performed within 48 h from admission |
| Marini et al., 2013 (Italy) (71) | Presence of arthralgia (TMJ pain at rest and during function, and on palpation, and crepitus) or OA (TMJ pain at rest and during function, and on palpation) | 1. Presence of myogenic pain  2. Presence of musculoskeletal pain  3. Co-occurring depressive disorders  4. Presence of odontogenic pain  5. Pregnant women  6. Malignancy  7. Systemic rheumatologic diseases |
| Masek et al., 1974 (Czech Republic) (72) | X | X |
| Murina et al., 2013 (Italy) (82) | X | X |
| Orefice et al., 2016 (Italy) (49) | 1. 18 to 55 years of age  2. < 1 year disease duration  3. 1.0 < EDSS < 3.5  4. ≥ 6 months IFN-β1a treatment  5. Experiencing IFN-β1a-related AEs | 1. MS-related conditions, such as current relapse  2. Past 30 days steroid therapy  3. Concomitant diseases precluding IFN-β1a  4. Pregnant or breastfeeding women  5. Cognitive decline  6. Pathological conditions interfering MS evolution  7. Allergy to NSAIDs  8. IFN-β1a intolerance |
| Ottaviani et al., 2019 (Italy) (63) | 1. 35 to 80 years of age  2. Non-smokers  3. NRS > 4 | 1. Signs or symptoms of injury or detectable pathologies in the  oral cavity  2. Periodontal disease  3. Past 3 months pain medications (anti-inflammatory or therapy  inducing xerostomia) |
| Palma et al., 2016 (Italy) (64) | 1. ≥ 18 years of age  2. ALSFRS-R score > 20  3. FVC > 30%  4. Treatment with rlz | 1. Other diseases of motor neurons  2. Participation to experimental treatments in the previous 3 months  3. Pregnant or breastfeeding women  4. Contraindications to the use of rlz  5. Patients undergoing tracheostomy, enteral or parenteral supply  6. Severe psychiatric disorders |
| Pickering et al., 2022  (Australia) (50) | 1. ≥ 18 years of age  2. Type 1 or type 2 diabetes with PNP  3. DN4 > 4  4. S-LANSS > 12  5. Ongoing anti-diabetic medications | 1. PNP due to hereditary sensory neuropathy, vitamin B12 or  folate deficiency, paraneoplastic diseases, advanced liver disease,  kidney disease, hypothyroidism, prolonged phenytoin, warfarin,  or immunosuppressive drug use  2. Ongoing herbal medications for pain relief  3. Pregnant or breastfeeding women, insufficient contraceptives  4. Alcohol or substance abuse  5. Allergy or sensitivity to PEA or any ingredients  6. Any clinically relevant abnormal findings |
| Rao et al., 2021 (Australia) (51) | 1. ≥ 18 years of age  2. PSQI > 5  3. Males and females  4. Females with child-bearing potential on a prescribed form of birth control  5. Agree not to change current diet or exercise or use other supplements for sleep disturbances for the study period | 1. Unstable or serious illness  2. Past 2 years malignancy or treatment for malignancy  3. Clinically significant inflammation connective tissue disease or arthritis  4. Current mood disorders  5. Current neurological disorders  6. OTC sleep medication or aid during the trial  7. Diagnosed sleep apnea  8. Diagnosed or consistent gastrointestinal issues disrupting sleep  9. Active smoking or nicotine, prescribed drug, or illegal substances  intake  10. Chronic past and/or present alcohol use  (>14 alcoholic drinks per week)  11. Regular stimulants intake (e.g. coffee, caffeine supplements,  or caffeine containing beverages) from midday onwards  12. Diagnosed clinical sleep disorder  13. Night-shift employment or other situations leaving one unable to  have a normal night’s sleep  14. Disturbed sleeping patterns caused by external factors (e.g.  children, partner, noise)  15. Allergic to the PEA or placebo formula  16. Pregnant or breastfeeding women or any condition  or non-medicated supplement making the participant  unsuitable for inclusion |
| Rao et al., 2023 (Australia) (76) | 1. Providing written informed consent  2. Patients agreeing not to take other supplements or medications aimed at preventing URTIs for the duration of the trial | 1. Cognitive damage  2. Serious mood disorders or neurological disorders  3. Unstable or serious illness  4. Acute sickness in the previous 2 months  5. Active smokers or nicotine/drugs abusers  6. Chronic alcohol use (>14 alcoholic drinks per week)  7. Allergy to any of the ingredients in the active or placebo formula  8. Pregnant or breastfeeding women  9. Medically prescribed medications that could affect the immune  and/or inflammatory response  10. Participated in a related clinical trial in the 1 month prior  11. Treatment for cancer, HIV, or the chronic use of steroids  in the past year |
| Rao et al., 2024 (Australia) (77) | 1. ≥ 18 years of age  2. Atopic dermatitis with redness, dry skin, scaling, and/or itchiness on their hands or arm  3. Providing written informed consent | 1. Active allergic skin responses  2. Pregnant or breastfeeding women  3. Current tobacco smoker  4. Chronic past and/or current alcohol use  5. Unstable or serious illness including renal, hepatic,  gastro-intestinal, cardiovascular, diabetes, mood disorders, cancer,  6. Using or used immunosuppressive medication within the last  3 months  7. Allergies to any of the ingredients in the Levagen+ or  comparator formula |
| Rossi et al., 2020 (Italy) (83) | 1. ≥ 18 years of age  2. Diagnosis of POAG/NTG  3. Controlled IOP (< 18 mmHg) with prostaglandin analogues monotherapy  4. Past 2 years stable IOP < 18 mmHg  5. Past 2 years stable disease (≤ -1 dB/year at MD of visual field)  6. Past 6 months no ocular surgery | 1. Ocular hypertension with normal optic nerve and visual field  2. Contraindication to PEA  3. Brimonidine eye drops use  4. Limiting conditions  5. Other causes of visual field changes |
| Salaffi et al., 2023 (Italy) (78) | 1. Providing written informed consent  2. 12-week dlx and pgb treatment | Other ongoing medications (except dlx and pgb) |
| Salehi et al., 2022 (Iran) (52) | 1. 18 to 60 years of age  2. Illness duration ≥ 2 years  3. PANSS negative ≥ 15  4. HDRS < 14  5. Clinical stability on rsp (PANSS total change ≤ 20% on 2 subsequent assessments within 2 weeks) | 1. IQ < 70  2. History of head trauma  3. Past 3 months history of ECT  4. Past 6 months substance or alcohol dependence  5. Pregnant or breastfeeding women  6. Suicidal ideation  7. History of neurosurgery  8. Current acute or chronic medical disease  9. History of allergy to rsp or PEA |
| Steels et al., 2018  (Australia) (53) | 1. NRS ≥ 4  2. Abstaining from any knee OA continuative medications | 1. Other forms of arthritis  2. Past 6 months joint injury  3. Past 30 days use of knee OA medications  4. BMI > 35  5. Pregnant or breastfeeding women  6. Uncontrolled diabetes  7. High cholesterol  8. Hypertension  9. Receiving anti-coagulation therapy  10. History of cerebrovascular accidents, stroke, or transient ischemia  11. MDD  12. Past 6 months unintended weight loss of > 15% body weight  13. Active substance abuse |
| Strobbe et al., 2013 (Italy) (84) | 1. Baseline IOP ≥ 22 mmHg for ≥ two measurements  2. Open anterior chamber angle at gonioscopy  3. C/D ratio < 0.4  4. Normal visual field parameters (MD < 3 dB and PSD < 2.5 dB)  5. Normal Glaucoma Hemifield Test  6. Normal corneal central thickness (530–560 lm) | 1. Cardiovascular disease or known cardiovascular risk factors  2. Taking vasoactive medications |
| Tartaglia et al., 2015 (Italy) (85) | 1. Untreated patients  2. 16 to 24 years of age | 1. Secondary dysmenorrhea  2. Chronic pelvic pain |
| Versace et al., 2023 (Italy) (54) | 1. Previous SARS-CoV-2 infection diagnosis (PCR testing of nasopharyngeal swab)  2. Subsequent infection recovery (2 consecutive negative PCR tests separated by ≥ 1 day)  3. Mild form of COVID-19, not necessitating inpatient admission  4. Complaints of fatigue and/or cognitive difficulties persisting after COVID-19 (FSS > 36, PCDS ≥ 1) | 1. Prior or concurrent neurological, psychiatric, endocrine,  metabolic, or cardiopulmonary conditions  2. Clinical and/or radiological evidence of active COVID-19 related  pneumonia  3. Anemia  4. Ongoing treatment with corticosteroids, antihistamines,  antihypertensives, diuretics, antidepressants, anxiolytics, hypnotics |
| Yuan et al., 2014 (China) (73) | 1. Mild to moderate Asteatotic eczema on the lower leg  2. Mild to moderate erythema, scaling, or dryness | 1. Active psoriasis or a history of psoriasis  2. Active allergic skin responses  3. Severe eczema |

<, less/smaller than; >, more/greater than; ≤, less/smaller than or equal to; ≥, more/greater than or equal to; ABC-C, Aberrant Behavior Checklist-Community; AD, Alzheimer's disease; AEs, Adverse Events; ALSFRS-R, ALS Functional Rating Scale-Revised; AP, Antipsychotic; BCVA, Best Corrected Visual Acuity; BMI, Body Mass Index; BOP, Bleeding on probing; C/D, Cup-to-disc; CAL, Clinical attachment level; COVID-19, SARS-CoV-2 infection; CP/CPPS, Chronic Prostatitis/Chronic Pelvic Pain syndrome; CPSI, Chronic Prostatitis Symptom Index; CTS, Carpal Tunnel Syndrome; DAS, Disease Activity Score; dlx, Duloxetine; DN4, Neuropathic pain diagnostic questionnaire; ECT, Electroconvulsive Therapy; EDSS, Expanded Disability Status Scale; EMS, Endometriosis; ENG, Electroneurography; FSS, Fatigue Severity Scale; FVC, Forced Vital Capacity; GCS, Glasgow Coma Scale; h, hours; HDRS, Hamilton Depression Rating Scale; IBD, Inflammatory Bowel Disease; IBS, Irritable Bowel Syndrome; IBS-C, IBS with predominant constipation; IBS-D, IBS with predominant diarrhoea; IBS-M, IBS with mixed symptoms; ICHD, The International Classification of Headache Disorders; IFN-β1a, Interferon-Beta1a; IOP, Intra-ocular pressure; IQ, Intelligence quotient; logMAR, Logarithm of the Minimal Angle of Resolution; lth, Lithium; MD, Mean Deviation; MDD, Major Depressive Disorder; NDD, Neurodegenerative Disorder; NIH, National Institutes of Health; NIHSS, National Institutes of Health Stroke Scale; NRS, Numeric Pain Rating Scale; NSAIDs, Nonsteroidal anti-inflammatory drugs; NTG, Normal Tension Glaucoma; OA, Osteoarthritis; OH, Ocular hypertension; OTC, Over-the-counter; PANSS, Positive and Negative Syndrome Scale; PCDS, Perceived Cognitive Difficulties Scale; PCR, Polymerase Chain Reaction; PD, Probing depth; PDis, Parkinson's disease; PEA, Palmitoylethanolamide; pgb, Pregabalin; POAG, Primary open glaucoma; PSD, Pattern standard deviation; PSQI, Pittsburgh Sleep Quality Index; rlz, Riluzole; rsp, Risperidone; S-LANSS, Self-reported Leeds Assessment of Neuropathic Symptoms and Signs; SCI, Spinal Cord Injury; TBI, Traumatic Brain Injury; TDI, Threshold, Discrimination and Identification subtests; TMJ, Temporo-mandibular joint; umPEA, Ultramicronized-PEA; URTI, Upper Respiratory Tract Infection; VAS, Visual Analogue Scale; YMRS, Young Mania Rating Scale.
